# Public health primary prevention implemented by clinical high-risk services for psychosis

**DOI:** 10.1038/s41398-022-01805-4

**Published:** 2022-01-28

**Authors:** Andrés Estradé, Gonzalo Salazar de Pablo, Alice Zanotti, Scott Wood, Helen L. Fisher, Paolo Fusar-Poli

**Affiliations:** 1 Early Psychosis: Interventions and Clinical-detection (EPIC) Lab, Department of Psychosis Studies, Institute of Psychiatry, Psychology & Neuroscience, King’s College London, London, UK; 2 Institute of Psychiatry and Mental Health, Department of Child and Adolescent Psychiatry, Hospital General Universitario Gregorio Marañón School of Medicine, Universidad Complutense, Instituto de Investigación Sanitaria Gregorio Marañón (IiSGM), CIBERSAM, Madrid, Spain; 3 Child and Adolescent Mental Health Services, South London and Maudsley NHS Foundation Trust, London, UK; 4 Department of Brain and Behavioral Sciences, University of Pavia, Pavia, Italy; 5 Yale School of Medicine, Yale University, New Heaven, CT USA; 6 King’s College London, Social, Genetic & Developmental Psychiatry Centre, Institute of Psychiatry, Psychology & Neuroscience, London, UK; 7 Economic & Social Research Council (ESRC) Centre for Society and Mental Health, King’s College London, London, UK; 8 National Institute for Health Research, Maudsley Biomedical Research Centre, South London and Maudsley NHS Foundation Trust, London, UK; 9 OASIS Service, South London and Maudsley NHS Foundation Trust, London, UK

**Keywords:** Schizophrenia, Bipolar disorder

## Abstract

Clinical High Risk for Psychosis (CHR-P) services have been primarily developed to support young people with attenuated symptoms (indicated prevention). No evidence-based appraisal has systematically investigated to what extent these clinics may implement other preventive approaches. PRISMA 2020-compliant systematic review of Web of Science, Cochrane Central Register of Reviews, and Ovid/PsychINFO, from inception until 14th June 2021, identifying original studies describing public health strategies: (a) service characteristics (configuration of mental health service, outreach, pathways to care); (b) universal interventions (general population); (c) selective interventions targeting CHR-P service-users or family/carers. Public health preventive initiatives were systematically stratified according to core social determinants of mental disorders associated with the 2030 Sustainable Development Goals promoted by the United Nations Member States (UN 2030 SDG) and good mental health outcomes. A total of 66 publications were included, providing data on 13 standalone, 40 integrated, three networks, and six regional or international surveys of CHR-P services across Europe, Asia, Oceania, Africa, North and South America, providing care to >28 M people. CHR-P services implement numerous public health initiatives targeting social and cultural (16 initiatives), economic (seven initiatives), demographic (six initiatives), environmental events (four initiatives) and neighbourhood (three initiatives) UN 2030 SGD determinants of mental disorders. There is additional evidence for CHR-P services promoting good mental health. The main barriers were the lack of resources for expanding public health prevention at a large scale. CHR-P services implement numerous public health prevention initiatives and promotion of good mental health beyond indicated prevention of psychosis.

## Introduction

A recent large-scale meta-analysis across 192 epidemiological studies (*n* = 708,561) found that 48.4% of mental disorders have their onset before age 18, with an overall peak at 14.5 years [1]. Efficacy of treatments is limited after the onset of mental disorders, leading to a high long-term burden upon individuals, families, healthcare systems and society more broadly [2]. Prevention in young people, therefore, represents a promising avenue to improve outcomes of mental disorders [3].

Primary prevention strategies may target the general population (i.e. universal); subgroups of people at higher-than-average risk of developing mental disorders (i.e. selective); or individuals with emerging or subthreshold manifestations of mental disorders (i.e. indicated) [4]. The Clinical High Risk for Psychosis paradigm (CHR-P) [5] indicated preventive model includes help-seeking adolescents or young adults (typically 14–35 years [6]) who accumulate risk factors for psychosis [7], often concurrent with functional [8] and neurocognitive impairments [9] and mild or infrequent symptoms of psychosis [5]. CHR-P individuals have a 25% increased risk of developing psychosis over the following 3 years, which is about 50-fold higher than in age-matched controls [10]. Specialised CHR-P services have been implemented in all six continents [11] to detect, formulate a prognosis (at risk or not at risk of transition to psychosis) and provide preventive care [6]. As an indicated preventive approach, CHR-P research has mostly focused on prevention of psychosis or other outcomes in young people presenting to these services. However, clinical care implemented by CHR-P services frequently encompasses public health initiatives that selectively target CHR-P individuals or their carers/family or universal interventions targeting the general population [12], as well as promotion of good mental health and wellbeing (as opposed to prevention of mental disorders [13]). Accordingly, CHR-P services may have the potential to address several potentially modifiable social determinants of psychosis risk, such as demographic factors (e.g. ethic related factors), economic factors (e.g. poverty), neighbourhood-related factors (e.g. social deprivation, infrastructure, urbanicity), exposure to traumatic environmental events (e.g. migration), violence (e.g. physical or emotional abuse) and natural/industrial disasters, and social and cultural factors such as education, social support, and social/cultural capital [14].

This potential has never been systematically appraised, highlighting a profound gap of knowledge. The primary aim of this systematic review is to provide an evidence-based systematic appraisal of public health initiatives implemented by CHR-P services worldwide, in the context of an established public health framework identifying social determinants of mental disorders [14], and complemented by good mental health domains [13].

## Methods

This systematic review was pre-registered (study protocol: PROSPERO CRD42020163640) and conducted in accordance with the “preferred reporting items for systematic reviews and meta-analyses” (PRISMA 2020) [15] guidelines.

### Search strategy and selection criteria

Three independent researchers (AE, GSdP and AZ) conducted the literature search and screening process for the identification of relevant articles. Discrepancies were resolved via consultation with a senior researcher (PFP). The search included the Web of Science database (Clarivate Analytics, incorporating the Web of Science Core Collection, BIOSIS Citation Index, KCI-Korean Journal Database, MEDLINE, Russian Science Citation Index, and SciELO Citation Index), Cochrane Central Register of Reviews, and Ovid/PsychINFO databases, from inception until 14th June 2021. The search terms used are appended in the eMethod 1. Titles and abstracts were screened, and potential full texts were assessed against inclusion and exclusion criteria. References of selected studies were screened for manual inclusion of any additional relevant publication.

Inclusion criteria were: (a) being an original study published in a scientific journal; (b) providing descriptive information of one or more CHR-P clinical services, as defined by established CHR-P assessment instruments (eMethod 2); and (c) providing information on different public health preventive interventions (defined as indicated below). Exclusion criteria were: (a) non-relevant designs such as secondary studies (i.e. reviews, meta-analyses, umbrella reviews), abstracts, conference proceedings, protocols, guidelines; (b) non-relevant populations, such as studies describing research other than CHR-P clinical services; and (c) no information on public health interventions. There was no restriction of language, and overlapping samples were not excluded.

### Measures and data extraction

Descriptive information for CHR-P services was systematically extracted by two independent researchers, and discrepancies were resolved via consultation with a senior researcher. Extracted information included: (i) general service information: name of CHR-P service, region and country, CHR-P age inclusion criteria, catchment area population, type of service/data (standalone, integrated, networks or surveys) [12]; and (ii) type of public health initiatives implemented (see below).

### Data synthesis

Public health initiatives were systematically clustered using a public health research framework focusing on core social determinants of mental disorders (Table 1). A large umbrella review addressed public health initiatives targeting social determinants of psychotic, bipolar and common mental disorders and empirically linked them with the 2030 Sustainable Development Goals (SDG) promoted by the United Nations Member States in 2015 [14] (UN 2030 SDG). Accordingly, public health initiatives were clustered across the following SDG domains: (i) demographic, (ii) economic, (iii) neighbourhood, (iv) environmental events, and (v) social and cultural domains. Within each of these domains, public health interventions were further stratified into three subcategories pertaining to (a) service characteristics (e.g. configuration of the mental health service, outreach, pathways to care); (b) universal interventions targeting the general population at a country, regional, city or neighbourhood level; and (c) selective interventions targeted to CHR-P service-users or family/carers but not primarily focusing on their presenting symptoms and risk for psychosis. As promotion of good mental health and wellbeing (as opposed to prevention, which is more concerned with avoiding mental disorders) is a core strategy of public health approaches, we additionally linked the public health interventions identified in the subcategories (b and c) to core good mental health domains. The latter were empirically validated in a previous consensus exercise (eTable 1): mental health literacy, attitudes towards mental disorders, self-perception and values, cognitive skills, academic/occupational performance, emotions, behaviours, self-management strategies, social skills, family and significant relationships, physical health, sexual health, meaning of life, and quality of life [13, 16].

**Table 1:** Essential public health interventions for social determinants of mental disorders associated with the UN 2030 Sustainable Development Goals (SDG), adapted from Lund 2018 [14].

Social determinants and description	Relevant SDGs	Distal and proximal social determinants of mental disorders	Potential public health interventions
**Demographic** *Demographic characteristics of populations that convey risk for, or protection from, mental illness*.	SDG 5: achieve gender equality and empower all women and girls.	Distal: community diversity, population diversity, longevity, survival. Proximal: age, ethnicity, gender.	Reduction of gender-based violence, child maltreatment, and racial discrimination and xenophobia.
**Economic** *Factors related to the production, consumption, and transfer of wealth that convey risk for, or protection from, mental illness*.	SDG 1: end poverty in all forms; SDG 2: end hunger and achieve food security; SDG 8: promote decent and sustainable work and economic growth; SDG 9: build resilient industry, innovation, and infrastructure; SDG 10: reduced inequalities within and among countries.	Distal: economic recession, economic inequality, macroeconomic policy. Proximal: income, debt, assets, financial strain, relative deprivation, unemployment, food security.	Cash transfers or basic income grants, reductions in income inequality, and improved employment.
**Neighbourhood** *features of an area or community that convey an increased risk, or protection from, mental illness*.	SDG 6: ensure access to clean water and sanitation; SDG 7: ensure access to affordable and clean energy; SDG 11: make cities and communities sustainable and safe; SDG 12: ensure responsible consumption and production patterns.	Distal: infrastructure, neighbourhood deprivation, built environment, setting. Proximal: safety and security, housing structure, overcrowding, recreational facilities/opportunities.	Improved housing, safe neighbourhoods.
***Environmental events*** *Serious events that disrupt a community’s ability to cope and convey an increased risk for mental illness*.	SDG 13: take urgent action to combat climate change and its impacts; SDG 16: promote peace, justice, and strong institutions.	Distal: natural disasters, industrial disasters, war or conflict, climate change, forced migration. Proximal: trauma, distress.	Reductions in violence, early response to environmental events, and action on protecting vulnerable ecosystems.
**Social and cultural** *Factors related to the organization of society, relationships, and social interactions that convey risk for, or protection from, mental illness*.	SDG 4: ensure inclusive and quality education for all.	Distal: community and social capital, social stability, cultural. Proximal: individual social capital, social participation, social support, education.	Improved education, strengthened social capital, and improving social support and networks for older adults.

## Results

### Study selection

**Fig. 1:**
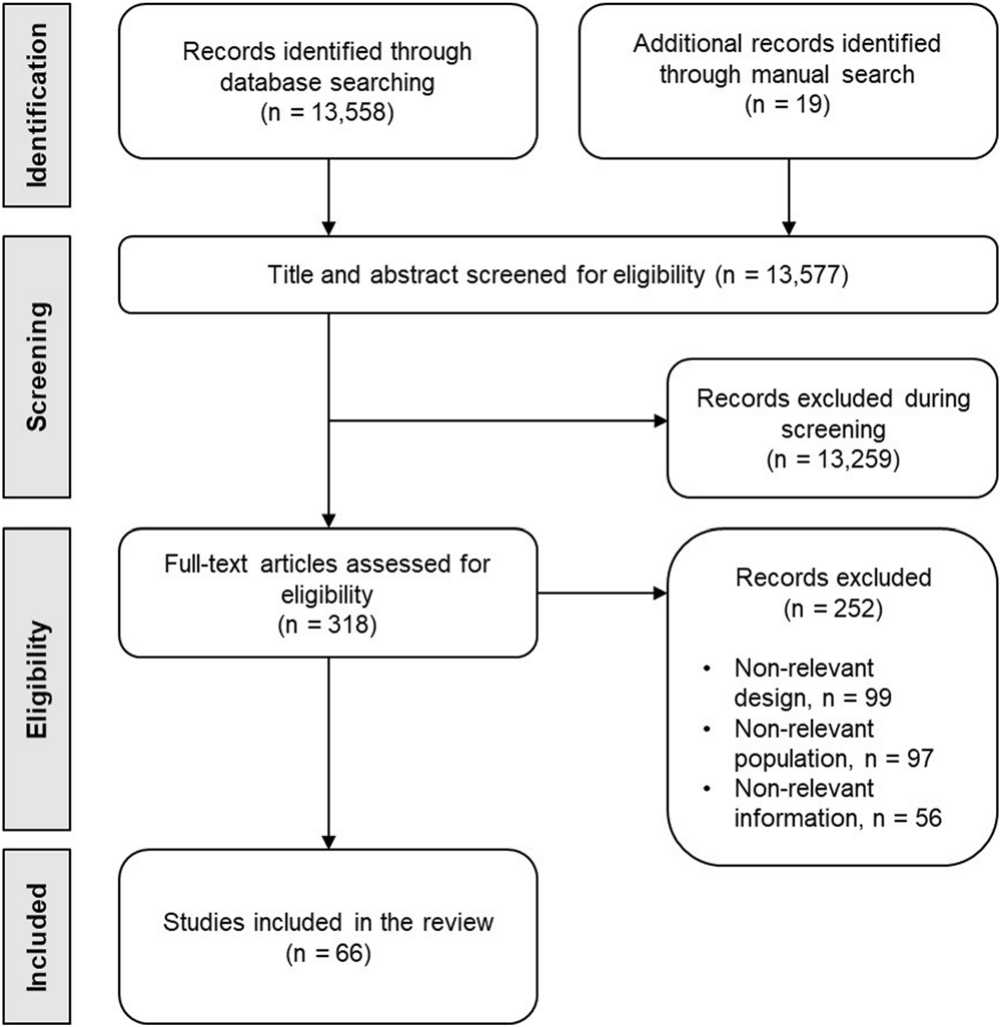
Study selection flow-chart. Preferred reporting items for systematic reviews and meta-analyses (PRISMA) flowchart outlining study selection process.

A total of 13,558 citations were screened for initial eligibility, and 318 full-text studies were evaluated. The final database consisted of 66 studies (Fig. 1). It included 13 standalone CHR-P services from Australia [17–20], Brazil [21], Germany [22], Netherlands [23], Poland [24, 25], South Korea [26], Singapore [27, 28], UK [29–36], and US [37, 38], and 40 integrated CHR-P services from Australia [39–42], Canada [43, 44], Chile [45], China (Hong Kong) [46–48], France [49], Greece [50, 51], Italy [52–61], Japan [62], Norway [63, 64], South Korea [65], Spain [66, 67], Switzerland [68–71], Tunisia [72], UK [36, 73, 74]; three networks of CHR-P services: the Early Detection Intervention and Prevention of Psychosis Program (EDIPPP) [75, 76], the Pan-London Network for Psychosis Prevention (PNP) [36], and Swiss Early Psychosis Project (SWEPP) [70]; five studies on national or regional surveys: 18 Early Intervention Services (EIS) from Canada [77], 45 [78] and 46 [79] EIS from Italy (two different surveys), 11 EIS from Portugal [80], and 50 EIS from the United Kingdom [81]; and one global survey of 47 CHR-P services [11] (eFig. 1). Overall, the CHR-P services included provided care to over 28 M people. Full details of the studies included in this review and CHR-P services are available in Table 2 and eTables 2–4.

**Table 2:** Description of CHR-P services included in the review.

Service(s)	Country – Region/City	CHR-P age inclusion*	Catchment area population
**Standalone CHR-P services**			
PACE [18–20]	Australia–Melbourne	14–30 years	NA
CASPAR [17]	Australia–Sydney	12–25 years	NA
ASAS [21]	Brazil–São Paulo	14–30 years	NA
FETZ Cologne [22]	Germany–Cologne	16–40 years	1,000,000
UMC EIS [23]	Netherlands–Utrecht	12–25 years	NA
PORT [24, 25]	Poland–Lodz	14–29 years	1,000,000
Seoul Youth Clinic [26]	South Korea–Seoul	15–35 years	NA
SWAP [27, 28]	Singapore	16–30 years	NA
PHK EIS [70]	Switzerland–Aargau	NA	NA
OASIS [29–36]	UK–London	14–35 years	1,358,646
THEDS [36]	UK–London	16–35 years	317,203
RAP [75, 76]	US–New York	NA	557,725
PIER [37, 38, 75, 76]	US–Portland	12–35 years	333,000
**Integrated CHR-P services**			
PAS [40]	Australia–Newcastle	NA	NA
EIS [39]	Australia–New South Wales	12–24 years	NA
SVH EPP [41, 42]	Australia–Melbourne	16–65 years	245,000
CAYR [43]	Canada–Montreal	14–35 years	1,900,000
PRIME [44]	Canada–Toronto	14–30 years	2,500,000
UCHIP [45]	Chile–Santiago	12–35 years	NA
EASY [46–48]	China–Hong Kong	12–25 years	7,000,000
C’JAAD [49]	France–Paris	15–30 years	NA
Eginition University Hospital EIS [50, 51]	Greece–Athens	15–40 years	NA
Catanzaro DMH EIS [52]	Italy–Catanzaro	17–30 years	35,000
Grosseto DMH EIS [52]	Italy–Grosseto	17–30 years	65,000
Rome (area D) DMH EIS [52]	Italy – Rome	17–30 years	250,000
Salerno DMH EIS [52]	Italy – Salerno	17–30 years	100,000
Programma 2000 [52, 56–58]	Italy–Milan	17–30 years	200,000
CCM2013 Project [53]	Italy–Lombardy, Tuscany and Liguria	15–24 years	NA
Ferrara DMH EIS [54]	Italy–Ferrara	15–35 years	360,000
Pr-EP [55]	Italy–Parma	12–35 years	500,000
ReARMS [59–61]	Italy–Reggio Emilia	13–35 years	﻿550,000
SAFE [62]	Japan–Sendai	14–35 years	1,060,000
POP [63, 64]	Norway–Stavanger	13–65 years	300,000
Mindlink [65]	South Korea–Gwangiu	15–30 years	1,500,000
ECEARP [66]	Spain–Barcelona	12–56 years	83,567
PAE-TPI [67]	Spain–Catalonia	18–35 years	NA
FEPSY [69, 70]	Switzerland–Basel	>18 years	200,000
EIS [70]	Switzerland–Basel-Bruderholz	NA	NA
FETZ Bern [70, 71]	Switzerland–Bern	8–40 years	1,000,000
JADE [70]	Switzerland–Geneva	18–25 years	NA
TIPP [70]	Switzerland–Lausanne	18–35 years	250,000
Station FP [70]	Switzerland–Münsterlingen	16–25 years	NA
FES [70]	Switzerland–Winterhur	16–35 years	NA
ZInEP Study [68, 70]	Switzerland–Zurich	13–35 years	1,300,000
CHiRP [72]	Tunisia–Tunis	NA	NA
EIS [73]	UK–Lincolnshire	14–65 years	750,000
HEADS UP [36]	UK–London	18–35 years	281,740
NEIS [36]	UK–London	18–35 years	353,245
Teesside EIP Service [74]	UK–Teesside	14–35 years	600,000
EDAPT [75, 76]	US–California	NA	466,488
M3P [75, 76]	US–Michigan	NA	344,791
EARLY [75, 76]	US–New Mexico	NA	661,422
EAST [75, 76]	US–Oregon	NA	631,853
**Surveys of EIS**			
Survey of 47 CHR-P services [11]	International–Africa, Asia, Europe, North America, South America, Oceania	NA	NA
Survey of 18 EIS [77]	Canada–Quebec	NA	≈3,750,000
Survey of 45 EIS [78]	Italy–Nationwide	NA	≈200,000 to 400,000
Survey of 46 EIS [79]	Italy–Nationwide	NA	NA
Survey of 11 EIS [80]	Portugal–Oporto, Santa Maria da Feira, Coímbra, Caldas da Rainha, Leiria, Lisbon, Faro	NA	3,400,000
Survey of 50 EIS [81]	UK–England	14–35 (85%)	NA

ASAS Evaluation and Follow-up of Adolescents and Young Adults in São Paulo, CASPAR Comprehensive Assessment Service for Psychosis and At Risk, CAYR Clinic for Assessment of Youth at Risk, CCM National Centre for Disease Prevention and Control, CHiRP clinical high-risk program of Razi Hospital, CHR-P clinical high-risk of psychosis, C’JAAD Evaluation Centre for Adolescents and Young Adults, DMH Department of Mental Health, EARLY Early Assessment and Resource Linkage for Youth, ECEARP Care Equipment for At-Risk of Psychosis Patients, EAST Early Assessment and Support Team, EASY Early Assessment Service for Young people with psychosis, EDAPT Early Detection and Preventive Treatment, EIP early intervention in psychosis, EIS early intervention service, FEPSY Basel early-detection-of-psychosis study, FETZ Early Recognition and Intervention Centre for Mental Crises, HEADS UP City & Hackney At-Risk Mental State Service, M3P Michigan Prevents Prodromal Progression, NA not available or unclear data, NEIS Newham Early Intervention Service, OASIS Outreach and Support in South London, PACE Personal Assessment and Crisis Evaluation clinic, PAE-TPI Early Psychotic Disorder Care Programmes, PAS Psychological Assistance Service, PHK Psychiatric Hospital Königsfelden, PIER Portland Identification and Early Referral, POP Prevention of Psychosis study, PORT Programme of Recognition and Therapy, Pr-EP Parma—Early Psychosis programme, PRIME, Toronto Prevention through Risk Identification, Management and Education, RAP Recognition and Prevention programme, ReARMS Reggio Emilia At-Risk Mental States programme, SAFE Sendai ARMS and first episode clinic, Station FP Psychiatric Hospital Münsterlinger Early Psychosis Outpatient Service, SVH EPP St Vincent’s Hospital early psychosis programme, SWAP Support for Wellness Achievement Programme, THEDS Tower Hamlets Early Intervention Service, TIPP Treatment and early Intervention in Psychosis Programme, UCHIP University of Chile High-risk Intervention Program, UK United Kingdom, UMC University Medical Centre, US United States, ZInEP Zurich Early Recognition Program. *General age inclusion criteria are reported if CHR-P-specific data is not available.

### Demographic domain

Demographic factors linked to an increased risk of mental disorders include distal factors, such as community and population diversity, longevity, and survival, and proximal factors, including age, gender, and ethnicity (Table 1) [14]. Related to this domain, the UN has postulated the achievement of gender equality and empowerment of all women by 2030 (SDG 5) [82]. These proximal factors were all targeted through CHR-P service characteristics (Table 3).

**Table 3:** Public health strategies delivered by CHR-P services targeting the social determinants of mental disorders, stratified by service characteristics, universal strategies, selective strategies, with the corresponding good mental health domains.

Domains	Interventions	
***Demographic***	***Service characteristics***	
	• Anti-stigma and youth-friendly community setting and service delivery. [17, 20, 35, 44, 52, 53, 65]	
	• Flexible outreach approach to enhance engagement of young people. [37, 65]	
	• Presence in catchment areas with high levels of ethnic minorities. [29, 30, 35, 76]	
	• Community engagement projects focused on young people from ethnic minorities. [32]	
	• Service-user involvement in service development and delivery to include multicultural backgrounds. [27, 32, 36, 77–79]	
	• Inclusiveness of LGBT+populations. [17]	
***Economic***	***Service characteristics***	
	• Presence in catchment areas with high levels of economic inequality, unemployment, and homelessness. [29, 30, 35]	
	• Recovery-oriented model of service delivery with focus on social and role functional support. [17, 27, 28, 30, 36, 51, 55, 59–61, 63, 64]	
	***Selective strategies and corresponding good mental health domains***	
	• Assessment of vocational history, goals and engagement, and cognitive functioning. [34, 71, 76]	*Academic/occupational performance, cognitive skills*
	• Occupational or supportive therapy on vocational/occupational functioning. [11, 34, 36, 42–44, 50–52, 55, 57, 58, 77–79]	*Academic/occupational performance*
	• Onsite vocational reintegration, including supported employment or IPS. [34, 35, 54, 55, 73, 76, 80]	*Academic/occupational performance*
	• Intensive networking with local/community stakeholders. [19, 28, 34, 38, 41, 74, 77]	*Academic/occupational performance*
	• Psychosocial support with housing and accommodation. [11, 19, 33, 74, 77]	*QoL*
***Neighbourhood***	***Service characteristics***	
	• Presence in catchment areas with high levels of social deprivation. [30, 35, 74]	
	• Presence in a prison setting. [30]	
	***Selective strategies and corresponding good mental health domains***	
	• Recreational therapy, activities or support. [43, 52, 53, 57, 58, 77]	*QoL, family and significant relationships*
***Environmental events***	***Service characteristics***	
	• Presence in catchment areas with high levels of refugees and asylum seekers. [29]	
	• Community engagement projects focused on asylum seekers and refugees. [32]	
	• Trauma-sensitive model of care. [39]	
	• Comprehensive assessment of lifetime exposure to traumatic events. [30, 67, 71]	
***Social and cultural***	***Universal strategies and corresponding good mental health domains***	
	• Mental health awareness and promotion campaigns for the general population, parents, and families. [19, 21, 22, 24, 25, 27, 29, 32, 36–38, 40, 44–49, 52, 56, 57, 62–64, 68–70, 75, 77, 79]	*Mental health literacy, attitude towards mental disorders*
	• Service-user involvement for the promotion of mental health literacy. [32, 36]	*Mental health literacy, attitude towards mental disorders*
	• Psychoeducation groups in collaboration with community organisations. [77]	*Mental health literacy, attitude towards mental disorders*
	• Education, awareness and anti-stigma campaigns for community organisations. [19, 20, 22, 27, 29, 30, 32, 36–38, 40, 43, 44, 53, 56, 57, 63, 66, 77–79]	*Mental health literacy, attitude towards mental disorders*
	• Training and mental health awareness for professionals working with young people. [19, 20, 22, 24, 27, 29, 32, 35–38, 40, 44, 45, 49, 50, 52, 54, 56, 57, 62–64, 68–70, 72, 73, 75, 77–79]	*Mental health literacy, attitude towards mental disorders*
	***Selective strategies and corresponding good mental health domains***	
	• Problem-solving training. [18, 57, 58, 78, 79]	*Self-management skills*
	• Individual or group psychoeducation. [18, 28, 31, 36, 49, 50, 58, 65, 71, 72, 74, 78–81]	*Mental health literacy, self-management skills*
	• Life/practical skills training. [74, 77]	*Self-management skills*
	• Family psychoeducation, counselling or support. [11, 17, 23, 26, 27, 32, 35, 36, 42, 43, 45, 49, 51, 52, 54–65, 71–73, 76–78, 81]	*Family and significant relationships*
	• Psychosocial support on social relationships and functioning. [11, 27, 28, 30, 36, 43, 45, 49, 50, 54, 55, 58, 59, 77]	*Social skills*
	• Educational support. [11, 27, 28, 35, 36, 49, 52, 57, 58, 74, 76, 77]	*Academic/occupational performance*
	• Cognitive Remediation Training [72]	*Cognitive skills*
	• Physical health assessment and monitoring. [36, 39, 41, 42, 45, 73, 80]	*Physical health, QoL*
	• Exercise/physical activity intervention, psychomotor therapy. [17, 23, 39, 43, 65, 77–79]	*Physical health, QoL*
	• Nutrition and healthy eating intervention or advice. [39, 43, 49, 65, 74, 77]	*Physical health, QoL*
	• Sleep hygiene or sleep interventions. [18, 30]	*Physical health, QoL*

IPS individual placement and support, LGBT+ lesbian, gay, bisexual, transgender+, QoL quality of life.

CHR-P services frequently adopted an anti-stigma and youth-friendly setting to increase the attractiveness of services and pathways to care for young people [17, 20, 35, 52, 65], typically opting for a community location far from main mental health hospitals or clinics and closer to public areas visited by young people [44, 53]. CHR-P services also implemented a flexible and community outreach approach to enhance access of young people, facilitating encounters outside standard clinical settings [37] or offering case-management via smartphone applications [65].

Likewise, CHR-P services were often located in catchment areas with a high proportion of ethnic minorities [29, 30, 35, 76], or implemented community engagement projects specifically targeting young people from black, Asian and other minority groups [32]. CHR-P samples varied greatly in terms of ethnical composition, ranging from >80% White in Italian [59–61] and Polish [25] services to non-White representing >30% of clinical samples in US services [37, 75, 76], >50% in the UK services [29, 31–34, 36], and up to 80% in Canadian samples [77], demonstrating that the paradigm is successful in reaching ethnically diverse audiences.

CHR-P services involved current and past service-users in developing and improving service delivery to young people with diverse cultural backgrounds [27, 32, 36, 77–79]. These activities encompassed the development of information material sensitive to youth culture [27], the creation of service-specific websites [36], the adaptation of service delivery to ethnically and culturally diverse populations [32], and the inclusion of peer support [77]. In terms of gender diversity, lesbian, gay, bisexual, transgender+ (LGBT+) populations represented 25.9% of the clinical samples in one Australian service [17], where efforts to further enhance and adapt service provision to this population are currently underway.

### Economic domain

Economic factors linked to increased risk of mental disorders range from macroeconomic, such as recession, inequality, and policy, to proximal factors including income, debt, assets, financial strain, relative deprivation, unemployment, and food security (Table 1) [14]. UN 2030 SDG linked to this domain include the eradication of poverty (SDG 1) and hunger (SDG 2), the promotion of sustainable growth and decent work conditions (SDG 8), resilient infrastructure, inclusive and sustainable industry, and innovation (SDG 9), and the reduction of inequalities (SDG 10) [82]. Economic factors were targeted by CHR-P via service characteristics and selective strategies (Table 3).

In terms of service characteristics, CHR-P services were at times located in catchment areas with high levels of unemployment, homelessness, and low socioeconomic status [29, 30, 35]. Lack of occupational activity among clinical samples in CHR-P services was not uncommon, ranging from 10–29% [25, 62, 76], to 30–40% [29–33, 39, 50, 55], and >40% [36, 45]. In addition, a recovery-oriented service model was frequently adopted by CHR-P services through the enhancement of social and academic/occupational functioning among young users [17, 27, 28, 30, 36, 51, 55, 59–61, 63, 64].

With respect to selective interventions, CHR-P services assessed vocational history, goals and engagement, and cognitive functioning as part of their standard operations [34, 71, 76]. Services also provided occupational or supportive therapy on vocational/occupational functioning, with interventions such as group activities (e.g. computer training), support job seeking and retention [11, 34, 36, 42–44, 50–52, 55, 57, 58, 77–79]. In addition, CHR-P services provided specific programmes for onsite vocational reintegration, such as supported employment or individual placement and support (IPS) [34, 35, 54, 55, 73, 76, 80], often through intense networking with local/community stakeholders [19, 28, 34, 38, 41, 74, 77].

Additional support extended to housing and accommodation via practical advice or coordination with housing services [11, 19, 33, 74, 77]. Homelessness among clinical samples of CHR-P services was reported at 3.0% in a UK service [32], and supported accommodation ranged from 3.3% [17] up to 17.5% [32]. Across different countries, such as Australia [17], France [49, 77], Japan [62], the UK [32], and Poland [25], most users live with their family or relatives, with only a minority living independently.

Through these initiatives, CHR-P services can impact academic/occupational performance, cognitive skills and quality of life and promote good mental health.

### Neighbourhood domain

Neighbourhood-related distal factors linked to an increased risk of mental illnesses include infrastructure, neighbourhood deprivation, built environment, and setting, and proximal factors include safety and security, housing structure, overcrowding, and access to recreational opportunities or facilities (Table 1) [14]. UN 2030 SDG include clean water and sanitation (SDG 6), access to affordable and reliable energy (SDG 7), sustainable, safe, and inclusive cities (SDG 11), and sustainable production and consumption (SDG 12) [82]. Neighbourhood-related factors have been targeted by CHR-P via service characteristics and selective strategies (Table 3).

In terms of service characteristics, CHR-P services have been located in neighbourhoods with high levels of social deprivation, including robbery, assault, substance use, and single people [30, 35, 74]. The proportion of single/unmarried users was most frequently in the ranges of >90% [25, 28, 45, 49, 52, 56, 57, 59, 61, 62, 76] or 80–90% [29–31, 33, 41], with levels of users without a steady relationship or partner in the range of 80–92% [22, 32, 36] (eTable 3). However, most studies do not discriminate between marital and interpersonal relationship status (i.e. with or without a steady relationship). In addition, the proportion of married CHR-P users appears generally lower than the national averages for the corresponding age categories (eTable 3), although these data have not been subjected to formal statistical comparison. This domain is also targeted by the presence of an assessment and detection team in prison for one UK service [30].

Selective strategies include recreational opportunities-related interventions, encompassing recreational therapy [77], social group activities (e.g. music, multimedia, expression) [52, 57, 58, 77], support in planning recreational activities [58], and engaging with different community organisations [53].

Through these activities, CHR-P services can impact the quality of life, family and significant relationships and promote good mental health.

### Environmental events domain

Exposures to natural and industrial disasters, war or conflict, forced immigration, and ecosystems hazards (e.g. floods, droughts) produced by climate change have been reported as distal risk factors of mental disorders, alongside the more proximal factors of trauma and distress (Table 1) [14]. The UN 2030 SDG postulate the need for urgent action to tackle climate change and its impacts (SDG 13) and the promotion of peace, justice, and strong institutions (SDG 16) [82]. CHR-P services addressed some of these environmental events by their service characteristics (Table 3).

Regarding conflict, war, and migration, a CHR-P service in the UK reported being located in a catchment area with a high proportion of refugees and asylum seekers [29]. The proportion of immigrants or non-native speakers among service users ranged from ≈10–20% in European and Australian services [17, 22, 39, 49, 55, 60, 66] to up to 80% in Canadian services [77]. Service delivery has been adapted to respond to traumatic exposures in different ways, either by conducting community engagement projects specifically targeting asylum seekers and refugees [32], or by adopting a trauma-sensitive model of care [39], and conducting comprehensive assessments of lifetime exposure to adverse events (e.g. sexual abuse, emotional abuse, physical abuse, and neglect) as part of regular clinical operations [30, 67, 71].

### Social and cultural domain

Social and cultural determinants of mental disorders encompass the distal factors of community, social and cultural capital, and social stability, and the proximal factors of individual social capital, participation and support, and education (Table 1) [14]. UN 2030 SDG linked to this domain include ensuring inclusive and equitable education for all (SDG 4) [82]. This domain was addressed by CHR-P services through universal or selective interventions (Table 3).

Within universal approaches, mental health awareness campaigns targeting the general population were commonly conducted at a community or city-wide level with the aim of improving mental health literacy, and reducing the stigma attached to mental disorders [19, 21, 22, 24, 25, 27, 29, 32, 36–38, 40, 44–49, 52, 56, 57, 62–64, 68–70, 75, 77, 79]. Activities varied greatly, including workshops, presentations, talks, tv or radio appearences [22, 27, 37, 44, 46–48, 68], participation in public events, fairs and exhibitions [27, 38, 40, 44, 46–48, 75], articles and publications in local newspapers and other printed or audio-visual media (e.g. videos, leaflets, brochures) [21, 22, 27, 37, 44, 46–48, 62–64, 69], and the use of online resources (e.g. service-specific website, social media) [29, 32, 36, 37, 46–48, 63, 64, 69, 70]. In Hong Kong, a new definition of “psychosis” was tested to improve a non-stigmatising engagement of the public [46–48]. On occasion, activities were conducted for targeted audiences, such as schools students, adolescents, or parents [24, 46–48, 57]. Other public awareness and education campaigns focused on specific community organisations or services [19, 20, 22, 27, 29, 30, 32, 36–38, 40, 43, 44, 53, 56, 57, 63, 66, 77–79], such as the development of a community preventive alliance alongside education, religious and other organisations [53]. Frequent community and public organisations targets encompassed: community healthcare services [20, 22, 29, 30, 32, 37, 40, 43, 44, 53, 56, 57, 66, 78, 79], social [32, 38, 56, 66], housing and employment [32] services, schools and colleges [20, 32, 37, 43, 44, 53, 57, 66, 78, 79], sports organisations [27], religious or faith centres [32, 37, 44, 53], multicultural groups [38, 53], charities and non-governmental organisations [29, 30, 32], wellbeing and youth centres [27, 32, 38, 40, 44, 57, 66], family services [27, 37], welfare organisations [27], local shops [32], libraries [44], governmental services [27], justice institutions [66], police and armed forces [27, 37, 44, 56], and correction facilities [37]. CHR-P services often collaborated with service users for the promotion of mental health literacy, for example, through the development of online resources based on their lived experiences [32, 36]. Psychoeducational groups in collaboration with community organisations (i.e. not restricted to patients) have also been employed [77].

Another universal approach implemented mental health awareness and training activities targeting professionals working in close contact with young people, commonly including GPs, mental health professionals, counsellors, private practitioners, teachers and educators, social workers, and front-line youth workers [19, 20, 22, 24, 27, 29, 32, 35–38, 40, 44, 45, 49, 50, 52, 54, 56, 57, 62–64, 68–70, 72, 73, 75, 77–79]. These activities addressed issues such as stigma reduction, risk factors for mental disorders, best preventive practices, case recognition, and were delivered through workshops [22, 24, 44, 68], education sessions and informal meetings [24, 32, 37, 38, 40, 56, 73, 75, 77], articles in professional journals and other printed media [19, 22, 37, 40, 62, 68], phone or email consultation [36, 62, 75], and websites specifically tailored to professionals [37]. Formal education courses for healthcare professionals, students and professionals from related areas were also implemented [19, 24, 27, 45, 49, 50, 70, 75, 77].

In terms of selective interventions, CHR-P services offered group problem-solving training [18, 57, 58, 78, 79], individual or group psychoeducation [18, 28, 31, 36, 49, 50, 58, 65, 71, 72, 74, 78–81], and life/practical skills training [74, 77]. Other initiatives focused on the familial and social network such as family psychoeducation, counselling and support, extensively provided by CHR-P services [11, 17, 23, 26, 27, 32, 35, 36, 42, 43, 45, 49, 51, 52, 54–65, 71–73, 76–78, 81]. Social functioning and relationships were promoted via psychosocial support, which included supportive counselling, case management for social functioning, and social group activities (e.g. social skills training, empowerment activities) [11, 27, 28, 30, 36, 43, 45, 49, 50, 54, 55, 58, 59, 77]. Other selective interventions promoting this good mental health domain included educational support [36, 52, 57, 58, 77], encompassing: case management aimed at educational functioning [11, 27, 28], collaboration with community organisations for optimising school reintegration and coordination [35, 49], facilitation of the Individualised Education Plans [76], and the identification of individualised educational programmes [74]. The cognitive skills domain was addresses via selective Cognitive Remediation Training interventions not primarily focusing on psychotic symptoms [72]. Finally, several CHR-P services implemented selective interventions to improve physical health and quality of life, such as routine physical health assessment and monitoring [36, 39, 41, 42, 45, 73, 80], exercise/physical activity intervention or psychomotor therapy [17, 23, 39, 43, 65, 77–79], nutrition and healthy eating intervention or advice [39, 43, 49, 65, 74, 77], and sleep hygiene/disturbances interventions [18, 30].

Through these activities, CHR-P services can impact mental health literacy, attitude towards mental disorders, self-management skills, family and significant relationships, social skills, academic/occupational performance, cognitive skills, physical health and quality of life, and promote good mental health.

## Discussion

This is the first systematic review to systematically appraise the evidence for public health interventions implemented by CHR-P services.

CHR-P services included in this review encompassed Europe, Asia, Oceania, Africa, and North and South America, providing care to over 28 M people in their respective catchment areas. CHR-P services were found to implement a wide range of public health approaches beyond indicated prevention through service characteristics, selective or universal interventions. The public health initiatives implemented by CHR-P services addressed core social determinants of mental disorders that align to the UN 2030 SDG [82], albeit to a variable degree. Public health initiatives implemented by CHR-P services targeted (in decreasing order of public health initiatives) social and cultural (16 initiatives), economic (7 initiatives), demographic (6 initiatives), environmental events (4 initiatives) and neighbourhood (3 initiatives) SGD determinants of mental disorders.

While the characteristics of these interventions have already been addressed above, it is important to highlight their close association with good mental health outcomes. Universal preventive interventions implemented by CHR-P services were strongly aimed at promoting mental health literacy and a positive attitude towards mental disorders, core dimensions of positive mental health [13], which are inversely related to stigma. Public stigma about mental disorders can lead to reduced help-seeking behaviour in young people and barriers to healthcare [83], reduced social networks, loneliness, fewer employment and housing opportunities, and an overall deterioration of mental state [84]. Accordingly, poor mental health literacy among the general population has been identified as a major barrier to the delivery of effective preventive interventions [26, 78, 79]. A recent meta-analysis found that psychoeducation through awareness and outreach campaigns in the general population is particularly effective to improve mental health literacy (ES = 0.69) [85]. Psychoeducation can help to disconfirm negative stereotypical beliefs about people with mental disorders and lead to positive attitudinal changes in the public, reducing stigma [86]. While psychoeducation represents a fundamental component of the needs-based intervention for CHR-P individuals [12], we demonstrate that CHR-P services extend its benefits to the general population, parents, families, community organisations and professionals working with young people, largely as part of their outreach campaigns. Notably, CHR-P service-users are frequently involved in customising these activities to the regional culture and sensitivity of young people [27, 32].

Selective interventions were mostly employed by CHR-P services to target the good mental health domains of self-management skills, social skills, family and significant relationships, academic/occupational performance, cognitive skills and overall quality of life. There is recent meta-analytic evidence supporting the efficacy of selective interventions for the promotion of quality of life (ES = 0.46), social skills (ES = 0.37), academic and occupational performance (ES = 0.21) in young people [85]. Physical health also represents a core good mental health domain addressed via a variety of selective interventions fostering healthy eating habits and physical activity, physical health monitoring, and sleep interventions. Improving physical health is of pressing urgency not only in early psychosis [39, 87, 88], but in several other mental disorders [89] and in the young general population [90]. Meta-analytic evidence supports the efficacy of selective (and universal) interventions for the promotion of good physical health among young people (ES = 0.285), with physical therapy, relaxation, and exercise being the most effective interventions [85]. Improving physical health is a tantalizing public health strategy, making sense for concurrently reducing the risk of many other mental disorders [91] such as psychotic, bipolar and depressive/anxiety disorders [92]. Strategically, the numerator of preventive cost and risk can be offset by a denominator of multiple preventable psychiatric and physical disease outcomes [93].

Taken together, the evidence reviewed indicates that CHR-P services are already implementing extensive public health preventive approaches beyond indicated intervention. This review supports the importance of increasing the roll out of these services across the globe and improved funding given the broader value and potential wider impact on population mental health of these services. At the same time, this review undertones the “prevention paradox” [94] argument, stating that as CHR-P services can only benefit a small minority of young people, they should be dismantled [95]. Indicated approaches are expected a priori to target the tip of the iceberg of the population-level risk and are thus complementary and not antithetical (as claimed [96]) to selective and universal approaches [4]. Future research and clinical practice should better integrate universal, selective and indicated approaches to synergistically and complementarily maximize their efficiency in young people [4]. A first step could be to overcome current barriers to mainstreaming public health interventions conducted by CHR-P services. The main barrier is the limited financial and political support for preventive services [21, 50, 52, 77, 81] linked to the lack of a preventive culture of mental health systems in some countries [78, 79]. A recent international survey of 47 CHR-P services indicated that 51.1% identified lack of financial support and 42.6% inadequate staffing resources as key implementation barriers [11]. For example, occupational and social workers, which are pivotal for public health initiatives, are present in less than half of CHR-P services [12], and even well-established CHR-P services find it hard to deliver comprehensive occupational interventions [34]. These challenges often translated into the difficulty of extending public health prevention to more isolated and rural areas [77]. In countries where sufficient funding has been made available, like Australia, youth-friendly preventive mental health services have been implemented at scale [97]. Refined CHR-P services, which broaden their remit to support young people experiencing a wide range of sub-clinical mental health symptoms (e.g. bipolar and depression beyond psychosis) and have public mental health and promotion remits, could become a model for providing preventive care in the general population [98]. A further barrier is that CHR-P public health research has been hindered by the lack of a unitary empirical framework. This study demonstrated that it is possible to adapt the public health framework [14] linked to the UN 2030 SDG [82] to CHR-P research. Our attempt provides a heuristic platform to facilitate further public health research in the CHR-P arena. Future research should specifically refine outreach approaches combining extensive community education within ethnically diverse communities and the creation of collaborative networks [32, 75, 99]. Additional work is also needed to customise effective public health prevention to LGBT+ populations [17] and culturally diverse and migrant populations [22, 45, 75]. Expanding initiatives to promote good mental health through anti-stigma campaigns [75], vocational and academic rehabilitation, and exercise and physical health [34, 45] is also a relevant point on the research agenda.

The main limitation of this review is that information on public health initiatives in CHR-P services is scattered and fragmented, preventing the feasibility of meta-analysis. Few studies [75] addressed public health initiatives upfront within their primary aims. Furthermore, some relevant data such as type of funding to CHR-P services were generally not reported in the original papers. In addition, conclusions are limited by the scarcity of standardised reports on the effectiveness of universal and selective strategies of CHR-P services for promoting good mental health domains. While the efficacy of these strategies has been systematically appraised in recent meta-analyses by our group [85, 100], there is no robust effectiveness evidence relating to CHR-P services. This may be due to the fact that research in this area is still emerging. We hope that the current systematic review, by providing an empirical classification of these initiatives will foster future research in this area. A further limitation is that there was minimal data available from low- and middle-income countries (studies from LMICs may be better represented in databases such as Lilacs, African Journals Online, and Global Health) and from countries where CHR-P services had more recently been introduced (e.g. Brazil, Tunisia, India, or Sweden) [11]. The level of research and evidence in LMICs is still modest and should become a mainstream topic on the future research agenda. In Latin America, EI services are in their early stages of development and mostly focused on FEP rather than CHR-P users [101, 102].

## Conclusion

CHR-P services implement a wide range of public health prevention initiatives and interventions for the promotion of good mental health beyond indicated prevention of psychosis. These initiatives address empirically validated social determinants of mental disorders, which align with the UN 2030 SDG.

## Supplementary information

Supplementary materials
