# Supplementary material for: Public health primary prevention implemented by clinical high-risk services for psychosis
Source: Transl Psychiatry. 2022 Jan 28;12:43. doi: 10.1038/s41398-022-01805-4 (PMC8799684; doi:10.1038/s41398-022-01805-4)
Supplement: Supplementary file 1 — Supplementary materials [file 41398_2022_1805_MOESM1_ESM.docx]

**SUPPLEMENTARY MATERIALS**

[**eMethod 1.** Search terms employed in the literature search 2](#_Toc92999532)

[**eMethod 2.** CHR-P assessment instruments 3](#_Toc92999533)

[**eTable 1.** Empirical domains of good mental health (adapted from Fusar-Poli et al. 2020^13^) 4](#_Toc92999534)

[**eTable 2.** Description of studies and characteristics of CHR-P service-users 5](#_Toc92999535)

[**eTable 3.** Marital status of CHR-P users and comparison with national level data 10](#_Toc92999536)

[**eTable 4.** Studies and specific type of public health interventions reported 13](#_Toc92999537)

[**eFigure 1.** Services, networks, and surveys included in the systematic review 20](#_Toc92999538)

[**eFigure 2.** Mental health literacy promotion materials at the OASIS service 21](#_Toc92999539)

# **eMethod 1.** Search terms employed in the literature search

The search terms employed in the literature search were: (“risk” OR “prodrom*” OR “ultra-high risk” OR “clinical high risk” OR “attenuat*” OR “high risk” OR “genetic high risk” OR “risk syndrome” OR “at risk mental state” OR “at-risk mental state” OR “ARMS” OR “risk of progression” OR “schizophrenia” OR “schizoaffective disorder” OR “schizophreniform disorder”) AND (“psychosis”) AND (“prevention” OR “intervention” OR “early intervention” OR “referral” OR “assessment” OR “service” OR “clinical service” OR “psychiatric service” OR “implementation” OR “care pathways” OR “mental health promotion” OR “public health”).

# **eMethod 2.** CHR-P assessment instruments

The presence of CHR-P individuals and related clinical service was defined by the use of the following assessment instruments, in line with several publications in this field:

Comprehensive Assessment of At-Risk Mental States (CAARMS^1^), Structured Interview for Psychosis-risk Syndromes (SIPS^2,3^), Bonn Scale for the Assessment of Basic Symptoms (BSABS^4^), Basel Screening Instrument for Psychosis (BSIP^5^), Schizophrenia Proneness Instrument^6^ - Adult (SPI-A) and Child and Youth (SPI-CY) version -, Positive and Negative Syndrome Scale (PANSS^7^), Scale for the Assessment of Negative Symptoms (SANS^8^), Brief Psychiatric Rating Scale (BPRS^9^) and Early Recognition Inventory (ERIraos^10^). The BPRS was included because the CAARMS (and the SIPS) derives from the BPRS; as such the BPRS was used in the earlier days to ascertain CHR-P individuals before the CAARMS was developed^11,12^.

# **eTable 1.** Empirical domains of good mental health (adapted from Fusar-Poli et al. 2020^13^)

| **Domain** | **Definition** |
| --- | --- |
| **Mental health literacy** | The ability to recognize and possess knowledge of a variety of different profiles of emerging and established mental disorders, factors and warning signs contributing to poor mental health as well as about the different mental health resources that can be accessed in case of need. |
| **Attitude towards mental disorder** | The way we react in front of someone with a mental disorder; Positive attitudes include understanding, being compassionate and empathic while decreasing discrimination towards people with poor mental health or with mental disorders. Negative attitude towards a person include stigma and stigmatizing behaviors, which leads to negative action or discrimination. |
| **Self-perceptions and values** | A collection of subjective beliefs, values and emotions about one’s own internal and external characteristics, shaping one’s attributional style, self-compassion and self-esteem, impacting awareness and acceptance, leading to living a valued life. |
| **Cognitive skills** | The ability to pay attention, remember and organize information, while having a degree of cognitive flexibility, attention to enable decision making and solve problems. |
| **Academic/occupational performance** | Objective learning and knowledge, study achievements, attendance and behavior as well as school adjustment and academic adaptation. |
| **Emotions** | Affective states with arousing or motivational properties that lead individuals to a certain response or behavior. |
| **Behaviors** | Behaviors are the conducts in which a person proceeds when a stimulus is presented to them. |
| **Self-management strategies** | Practical, everyday skills needed to effectively and independently take care of oneself and to function and meet the demands of the environment: coping skills to deal with stress, problem solving and decision making to face the adversities that may appear. |
| **Social skills** | Social skills are abilities that allow young people to interact and communicate with each other to foster positive relations. |
| **Family and significant relationships** | The ability to establish meaningful relationships with other family members; healthy and positive relationships and connectedness with family members within a secure environment, which facilitates positive communication and interaction. |
| **Physical health** | Physical variables, visible symptoms and measures related to a positive physical status and dimensions of health. |
| **Sexual health** | A state of physical, emotional, mental and social well-being in relation to sexuality. |
| **Meaning of life** | Feeling that life has a purpose and a significance |
| **Quality of life** | The general well-being of a person, defined in terms of health, happiness, and satisfaction with life. |

# **eTable 2.** Description of studies and characteristics of CHR-P service-users

| Author, year | Services included in publication; Country/Region | Type of service/data | Ethnic distribution, nationality, or minority status^a^ | Occupational activity or SES | Housing/living conditions |
| --- | --- | --- | --- | --- | --- |
| Adamson, 2018^14^ | Lincolnshire EIS; UK | Integrated | 83.9% White, 0.8% Black, 1.0% Asian, 1.0% Mixed, 13.2% unknown* | NA | NA |
| Addington, 2008^15^ | PRIME; Canada | Integrated | NA | NA | NA |
| Belvederi Murri, 2020^16^ | Ferrara DMH EIS; Italy | Integrated | NA | NA | NA |
| Bertulies-Esposito, 2020^17^ | 17 EIS (6 services with CHR-P component); Quebec - Canada | Regional survey | 0-80% visible minorities; 0-80% first or second-generation immigrant** | 20-95% studying at admission; 50% working at admission** | Living with family: 60-95%; living independently: 0-40%** |
| Broome, 2005^18^ | OASIS; UK | Standalone | 38.6% White British, 24.5% Caribbean and African, 15.8% Black British, 8.8% other White, 3.5% Mixed, 3.5% Asian Oriental, 3.5% Middle East, 1.8% Asian Indian | 69.1% occupied (38.2% employed, 30.9% student), 30.9% unemployed | NA |
| Carr, 2000^19^ | PAS; Australia | Integrated | NA | NA | NA |
| Coates, 2019^20^ | New South Wales EIS; Australia | Integrated | 9.02% Aboriginal and Torres Straits Islander** | 30.3% employed, 30% studying, 40% neither in employment nor education** | NA |
| Cocchi, 2013^21^ | Programma2000; Italy | Integrated | 93% Italian, 1% European non-Italian, 6% non-European | NA | NA |
| Cocchi, 2015a^22^ | Catanzaro DMH EIS, Grosseto DMH EIS, Rome (area D) DMH EIS, Salerno DMH EIS, Programma 2000; Italy | Integrated | 91% Italian, 4% European non-Italian, 4% non-European | NA | NA |
| Cocchi, 2015b^23^ | 45 EIS services (29 services with CHR-P component); Italy | National survey | NA | NA | NA |
| Coentre, 2020^24^ | 11 EIS (8 services with CHR-P component); Portugal | National survey | NA | NA | NA |
| Formica, 2020^25^ | PACE, Australia | Standalone | NA | NA | NA |
| Fusar-Poli, 2013^26^ | OASIS; UK | Standalone | 52.1% White, 35.1% Black, 5.2% Asian, 5.9% Mixed, 1.7% other | 54.4% occupied (27.5% employed, 26.9% student) | NA |
| Fusar-Poli, 2014^27^ | OASIS; UK | Standalone | 40.8% White British, 34.1% Black, 11.4% White other, 13.7% other | 55.5% occupied (29.1% employed, 26,4% student); 44.5% unemployed | NA |
| Fusar-Poli, 2019^28^ | PNP network^a^; UK | Network of CHR-P services | 42.5% White, 16.7% Asian, 8.2% Black African, 5.8% Black Caribbean, 14.6% Black British, 12.3% other | 58.9% unemployed, 41.1% studying or employed | NA |
| Fusar-Poli, 2020^29^ | OASIS; UK | Standalone | 46.44% White, 32.37% Black, 7.12% Asian, 14.07% other | 39.22% unemployed | 49.55% ﻿lived with their own family, 26.02% lived in a rental house, 17.47% supported accommodation, 3.03% homeless, 1.25% lived owner |
| Gaspar, 2018^30^ | UCHIP; Chile | Integrated | NA | 3.7% employed | NA |
| Ghio, 2012^31^ | 46 EIS (26 services with CHR-P component); Italy | National survey | NA | NA | NA |
| Green, 2011^32^ | OASIS; UK | Standalone | 40% White British, 17.9% White others, 27.4% Black, 13.7% others, 1.7% missing | 68.4% occupied (employed 36.8%, student 31.6%), 29.9% unemployed | NA |
| Janssen, 2020^33^ | UMC EIS; Netherlands | Standalone | NA | NA | NA |
| Joa, 2015^34^ | POP; Norway | Integrated | NA | NA | NA |
| Joa, 2021^35^ | POP; Norway | Integrated | NA | NA | NA |
| Katsura, 2014^36^ | SAFE; Japan | Integrated | NA | 84.9% occupied (72.6% student, 8.5% employed, 3.8% housewife), 15.1% unemployed | 90.1% living with others |
| Kim, 2020^37^ | Mindlink; South korea | Integrated | NA | NA | NA |
| Kollias, 2016^38^ | Eginition University Hospital EIS; Greece | Integrated | NA | 42.3% employed, 30.8% unemployed, 26.9% student | NA |
| Kollias, 2020^39^ | Eginition University Hospital EIS; Greece | Integrated | NA | NA | NA |
| Kotlicka-Antczak, 2015^40^ | PORT; Poland | Standalone | NA | NA | NA |
| Kotlicka-Antczak, 2016^41^ | PORT; Poland | Standalone | 100% White | 20.2% working, 79.8% not working, 13.13% no educational or vocational activity at entry, 10.1% educational or vocational dropout | 5.05% alone, 83.84% with parents, 2.02% with sibling, 3.03% with spouse, 4.04% with other relatives, 1.01% with partner, 1.01% in therapeutic centre |
| Kotlicka-Antczak, 2020^42^ | 47 CHR-P services; Western Europe, East Asia, and North America | International survey | NA | NA | NA |
| Kwon, 2012^43^ | Seoul Youth Clinic; South Korea | Integrated | NA | NA | NA |
| Leuci, 2019^44^ | Pr-EP; Italy | Integrated | 79% Italian** | 33% unemployed, 28.8% students** | NA |
| Louza, 2008^45^ | ASAS; Brazil | Standalone | NA | 100% unemployed | NA |
| Lynch 2016^46^ | EDIPP network^b^; US | Network of CHR-P services | 62% White, 11% > 1 race, 9% African American, 7% other, 4% Asian, 4% missing, 1% American Indian, 1% Native Hawaiian | NA | NA |
| McFarlane, 2012^47^ | EDIPP network^b^; US | Network of CHR-P services | 61% White, 17% Hispanic, 8% African American, 4% Asian American | 84% in school or working | NA |
| McFarlane, 2010^48^ | PIER; US | Integrated | 6% African American, 2% Asian American, 2% Latino American, 1% was Native American, 2% other racial-ethnic groups, 87% European American | NA | NA |
| Meneghelli, 2010^49^ | Programma2000; Italy | Integrated | NA | NA | NA |
| Meneghelli, 2020^50^ | Programma2000; Italy | Integrated | NA | NA | NA |
| Michel, 2021^51^ | FETZ-Bern; Switzerland | Integrated | 89.8% Swiss | 89.8% employed | NA |
| Oppetit, 2016^52^ | C’JAAD; France | Integrated | 91.8% native French language | NA | 21.9% living alone, 71.2% with parents, 6.9% other |
| Ortega, 2020^53^ | PAE-TPI; Spain | Integrated | NA | NA | NA |
| Parabiaghi, 2019^54^ | CCM2013 Project; Italy | Integrated | NA | NA | NA |
| Pelizza, 2019^55^ | ReARMS; Italy | Integrated | 87.3% White | NA | NA |
| Pelizza, 2020^56^ | ReARMS; Italy | Integrated | 91.1% White;  86.7% Italian | NA | NA |
| Penno, 2017^57^ | SVH EPP; Australia | Integrated | NA | NA | NA |
| Phillips, 2002^58^ | PACE; Australia | Standalone | NA | NA | NA |
| Poletti, 2020^59^ | ReARMS; Italy | Integrated | 86.4% White | NA | NA |
| Power, 2007^60^ | OASIS; UK | Standalone | NA | NA | NA |
| Pruessner, 2015^61^ | CAYR; Canada | Integrated | NA | NA | NA |
| Quijada, 2010^62^ | ECEARP; Spain | Integrated | 20% immigrant | 20% low-low, 20% low, 20% middle-low, 25% middle, 15% middle-high SES | NA |
| Rao, 2013^63^ | SWAP; Singapore | Standalone | NA | NA | NA |
| Riecher-Rossler, 2007^64^ | FEPSY; Switzerland | Integrated | NA | NA | NA |
| Ruff, 2012^65^ | PIER; US | Standalone | NA | NA | NA |
| Schultze‑Lutter, 2009^66^ | FETZ; Germany | Standalone | 88% German | 22.9% unemployed | NA |
| Selvendra, 2014^67^ | SVH EPP; Australia | Integrated | NA | NA | NA |
| Simon, 2012^68^ | SWEPP network^c^; Switzerland | Network of CHR-P services | NA | NA | NA |
| Stain, 2017^69^ | 50 EIS (53% with CHR-P component); UK | National survey | NA | NA | NA |
| Tang, 2010^70^ | EASY; Hong Kong | Integrated | NA | NA | NA |
| Tay, 2015^71^ | SWAP; Singapore | Standalone | 79.4% Chinese, 12.3% Malay, 5.8% Indian, 2.5% other | 22.6% employed, 41.9% student, 26.5% national service freshman, 0.6% housewife, 1.3% other, 7.1% unemployed | NA |
| Theodoridou 2014^72^ | ZInEP; Switzerland | Integrated | NA | NA | NA |
| Tiffin, 2007^73^ | Teesside EIP Service; UK | Integrated | NA | NA | NA |
| Tognin, 2019^74^ | OASIS; UK | Standalone | 44.3% White, 28% Black, 10% Asian, 17.1% other | NA | NA |
| Ventura, 2021^75^ | CHiRP; Tunisia | Integrated | NA | NA | NA |
| Wong, 2008^76^ | EASY; Hong Kong | Integrated | NA | NA | NA |
| Wong, 2012^77^ | EASY; Hong Kong | Integrated | NA | NA | NA |
| Yang, 2020^78^ | CASPAR; Australia | Standalone | 89.1% Australian born; 90.2% English as 1st language; 17.4% culturally and linguistically diverse; 25.9% LGBT+*** | NA | 89.1% living at home, 7.6% living independently, 3.3% subsidized accommodation*** |
| Yung, 1998^79^ | PACE, Australia | Standalone | NA | NA | NA |

*Legend: ASAS, Evaluation and Follow-up of Adolescents and Young Adults in São Paulo; CASPAR, Comprehensive Assessment Service for Psychosis and At Risk; CAYR, Clinic for Assessment of Youth at Risk; CCM, National Centre for Disease Prevention and Control; CHiRP, clinical high-risk program of Razi Hospital; CHR-P, clinical high-risk of psychosis; C’JAAD, Evaluation Centre for Adolescents and Young Adults; EASY, Early Assessment Service for Young people with psychosis; DMH, Department of Mental Health; ECEARP, Care Equipment for At-Risk of Psychosis Patients; EIP, early intervention in psychosis; EIS, early intervention service; FEPSY, Basel early-detection-of-psychosis study; FETZ, Early Recognition and Intervention Centre for Mental Crises; LGBT+, lesbian, gay, bisexual, transgender+; NA, not available or unclear data; OASIS, Outreach and Support in South London; PACE, Personal Assessment and Crisis Evaluation clinic; PAE-TPI, Early Psychotic Disorder Care Programmes; PAS, Psychological Assistance Service; PIER, Portland Identification and Early Referral; POP, Prevention of Psychosis study; PORT, Programme of Recognition and Therapy; Pr-EP, Parma—Early Psychosis programme; PRIME, Toronto Prevention through Risk Identification, Management and Education; ReARMS, Reggio Emilia At-Risk Mental States programme; SAFE, Sendai ARMS and first episode clinic; SES, socio-economic status; SVH EPP, St Vincent’s Hospital early psychosis programme; SWAP, Support for Wellness Achievement Programme; UCHIP, University of Chile High-risk Intervention Program; UK, United Kingdom; UMC, University Medical Centre; US, United States; ZInEP, Zurich Early Recognition Program. ^a^ Pan-London Network for Psychosis- Prevention (PNP) includes the following services: Outreach and Support in South London (OASIS), City & Hackney At-Risk Mental State Service (HEADS UP), Newham Early Intervention Service (NEIS), Tower Hamlets Early Intervention Service (THEDS). ^b^Early Detection, Intervention and Prevention of Psychosis Program (EDIPPP) includes the following services: Early Assessment and Resource Linkage for Youth (EARLY); Early Detection and Preventive Treatment (EDAPT); Early Assessment and Support Team (EAST); Michigan Prevents Prodromal Progression (M3P); Portland Identification and Early Referral (PIER); Recognition and Prevention programme (RAP). ^c^The Swiss Early Psychosis Project (SWEPP) national network includes the following services: Bruderholz EIS, Basel early-detection-of-psychosis study (FEPSY), FES Psychiatric Services Winterthur, Early Recognition and Intervention Centre for Mental Crises (FETZ-Bern), JADE University Hospital of Geneva, Psychiatric Hospital Königsfelden EIS, Station FP, Treatment and early Intervention in Psychosis Programme (TIPP), Zurich Early Recognition Program (ZInEP), Secteur Psychiatrique Nord Yverdon-les-Bains, Secteur Psychiatrique de l’Ouest Nyon/Prangins, Intervention Précoce dans les Troubles Pscyhotiques.*

*^a^The sum of all values might exceed 100 as the subgroups can be overlapping.*

**Sociodemographic characteristics of all assessed patients referred to the service.*

***Sample combines ultra-high risk for psychosis and first-episode of psychosis service-users.*

****Sample combines ultra-high risk for psychosis and anxiety, depression, and personality disorders service-users.*

# **eTable 3.** Marital status of CHR-P users and comparison with national level data

| Author, year | Services included in publication; Country/Region | Gender (% male) in CHR-P sample | Mean age of CHR-P sample in years (SD) | Marital status of CHR-P users | National-level proportion of individuals currently married by corresponding service inclusion criteria | Source of national level data* |
| --- | --- | --- | --- | --- | --- | --- |
| Brazil | | | | | | |
| Louza, 2008^45^ | ASAS | 33.3 | 21.7 (4.4), range | 72.2% single, 22.2% married, 5.6% divorced | 15-19 years: 4.2% male, 15.1% female  **20-24 years: 27.3% male, 42.1% female**  25-29 years: 52.2% male, 61% female | 2010 Census |
| Chile | | | | | | |
| Gaspar, 2018^30^ | UCHIP | 70.3 | 17.6 (2.9), range 12-28 | 100% single | **15-19 years: 3% male, 5.7% female**  20-24 years: 16.3% male, 23.2% female  25-29 years: 37.1% male, 45.2% female  30-34 years: 56.9% male, 56.1% female | 2011 Annual CASEN |
| Germany | | | | | | |
| Schultze‑Lutter, 2009^66^ | FETZ | 61.6 | 24.8 (6.1) | 91.5% single, 5% married/steady partner, separated/ divorced/ widowed 3.5% | 15-19 years: 0.1% male, 0.7% female  20-24 years: 3.9% male, 10.9% female  **25-29 years: 18.7% male, 32.7% female**  30-34 years: 38.5% male, 52.5% female  35-39 years: 52.5% male, 62.8% female | 2004 Estimate |
| Greece | | | | | | |
| Kollias, 2016^38^ | Eginition University Hospital EIS | 53.8 | 25.3 (4.3) | 73.1% single, 23.1% married | 15-19 years: 0.6% male, 1.8% female  20-24 years: 4.5% male, 13.9% female  **25-29 years: 17.2% male, 36.7% female**  30-34 years: 43.5% male, 63% female  35-39 years: 63.9% male, 75.2% female | 2011 Census |
| Italy | | | | | | |
| Meneghelli, 2010^49^ | Programma2000 | 70.4 | 22.3 (3.6) | 98.8% single | 15-19 years: 0% male, 0.5% female  **20-24 years: 2.1% male, 9.3% female**  25-29 years: 14.5% male, 32.4% female | 2009 Estimate |
| Cocchi, 2015a^22^ | Catanzaro DMH EIS, Grosseto DMH EIS, Rome (area D) DMH EIS, Salerno DMH EIS, Programma 2000 | 79 | 21.2 (4.7) | 100% unmarried |  |  |
| Cocchi, 2013^21^ | Programma2000 | 68 | 22.1 (3.6) | 99% unmarried | 15-19 years: 0% male, 0.5% female  **20-24 years: 1.9% male, 8.8% female**  25-29 years: 13.9% male, 31.3% female | 2010 Estimate |
| Japan | | | | | | |
| Katsura, 2014^36^ | SAFE | 37.7 | 20.0 (4.3) | 95.3% unmarried | 15-19 years: 0.3% male, 0.8% female  **20-24 years: 6.2% male, 10.4% female**  25-29 years: 27.4% male, 38.3% female  30-34 years: 50.6% male, 62.8% female | 2015 Census |
| Poland | | | | | | |
| Kotlicka-Antczak, 2016^41^ | PORT | 45.5 | 18.97 (3.56), range 15-32 | 95.96% single, 3.03% married | **15-19 years: 0.2% male, 1.2% female**  20-24 years: 8.1% male, 21.1% female  25-29 years: 40.1% male, 58.7% female | 2011 Census |
| United Kingdom | | | | | | |
| Broome, 2005^18^ | OASIS | 65.5 | 24.1 (4.165) | 89.3% never married, 7.2% married/living with partner, 3.6% separated or divorced | 15-19 years: 1.6% male, 4.6% female  **20-24 years: 16.5% male, 29.6% female**  25-29 years: 47% male, 57.1% female  30-34 years: 65.6% male, 68.3% female | 2001 Census |
| Green, 2011^32^ | OASIS; | 57.3 | 23.7 (4.7) | 83.5% single, 12% partner | 15-19 years: 1.1% male, 2.9% female  **20-24 years: 14.9% male, 25.9% female**  25-29 years: 43.5% male, 53.6% female  30-34 years: 63.9% male, 70.5% female | 2011 Census |
| Fusar-Poli, 2013^26^ | OASIS | 56.1 | 22.9 (4.61) | 81.5% single, 13.6% married/living with partner, 4.9% separated/divorced |  |  |
| Fusar-Poli, 2014^27^ | OASIS | 56.6 | 22.9 (4.5) | 83.1% single, 8.6% living together, 4.3% divorced/separated, 3.9% married |  |  |
| Fusar-Poli, 2019^28^ | PNP network^a^ | 58.1 | Mean age (SD) across services in PNP: 22.84 (4.93), 22.01 (4.76), 22.02 (4.02), 23.35 (4.88), 22.33 (3.66). | 17.6% married or with partner, 82.4% single |  |  |
| Fusar-Poli, 2020^29^ | OASIS | 55.33 | 22.6 (4.9), range 13-36 years | 79.59% single, 14.07% in a relationship, 4.12% married, 2.23% separated or divorced |  |  |
| Singapore | | | | | | |
| Tay, 2015^71^ | SWAP | 69.7 | 21.0 (SD: 3.5), range 15-31 | 96.8% single or never married, 3.2% married | 15-19 years: 0.1% male, 0.4% female  **20-24 years: 2.2% male, 7.5% female**  25-29 years: 24.7% male, 44.2 female | 2010 Census |

*Legend: National level marital status data was retrieved from:* [*https://population.un.org/MarriageData/index.html#/maritalStatusData*](https://population.un.org/MarriageData/index.html#/maritalStatusData) *(accessed on November 2021). We report age categories corresponding to the service’s age inclusion criteria. In bold we highlight the age category closest to the CHR-P sample mean age. ASAS, Evaluation and Follow-up of Adolescents and Young Adults in São Paulo; CASEN, National Socio-Economic Characterization Survey; CHR-P, clinical high-risk of psychosis; DMH, Department of Mental Health; EIS, early intervention service; FETZ, Early Recognition and Intervention Centre for Mental Crises; OASIS, Outreach and Support in South London; PORT, Programme of Recognition and Therapy; SAFE, Sendai ARMS and first episode clinic; SVH EPP, St Vincent’s Hospital early psychosis programme; SWAP, Support for Wellness Achievement Programme; UCHIP, University of Chile High-risk Intervention Program. ^a^ Pan-London Network for Psychosis- Prevention (PNP) includes the following services: Outreach and Support in South London (OASIS), City & Hackney At-Risk Mental State Service (HEADS UP), Newham Early Intervention Service (NEIS), Tower Hamlets Early Intervention Service (THEDS).*

** Available data closest to the recruitment endpoint if reported. If recruitment period not specified, available data closest to publication date was selected.*

# **eTable 4.** Studies and specific type of public health interventions reported

| Author, year | Specific type of public health initiatives reported |
| --- | --- |
| Adamson, 2018^14^ | Family psychoeducation, counselling or support  Onsite vocational reintegration, including supported employment or IPS  Physical health assessment and monitoring  Training and mental health awareness for professionals working with young people |
| Addington,2008^15^ | Anti-stigma and youth-friendly community setting and service delivery  Education, awareness and anti-stigma campaigns for community organisations  Mental health awareness and promotion campaigns for the general population, parents, and families  Occupational or supportive therapy on vocational/occupational functioning  Training and mental health awareness for professionals working with young people |
| Bertulies-Esposito, 2020^17^ | Diet and healthy eating intervention or advice  Drama, music/arts therapy  Education, awareness and anti-stigma campaigns for community organisations  Exercise/physical activity intervention, psychomotor therapy, access to local gym  Family psychoeducation, counselling or support  Intensive networking with local/community stakeholders  Life/practical skills training  Mental health awareness and promotion campaigns for the general population, parents, and families  Occupational or supportive therapy on vocational/occupational functioning  Psychoeducation groups in collaboration with community organisations  Psychosocial support on social relationships and functioning  Educational support  Psychosocial support with housing and accommodation  Recreational therapy, activities or support  Service-user involvement on service development and delivery to include multicultural backgrounds  Training and mental health awareness for professionals working with young people |
| Broome, 2005^18^ | Education, awareness and anti-stigma campaigns for community organisations  Mental health awareness and promotion campaigns for the general population, parents, and families  Presence in catchment area with high levels of economic inequality, unemployment, and homelessness  Presence in catchment area with high levels of ethnic minorities  Presence in catchment area with high levels of refugees and asylum seekers  Training and mental health awareness for professionals working with young people |
| Carr, 2000^19^ | Education, awareness and anti-stigma campaigns for community organisations  Mental health awareness and promotion campaigns for the general population, parents, and families  Training and mental health awareness for professionals working with young people |
| Coates, 2019^20^ | Diet and healthy eating intervention or advice  Exercise/physical activity intervention, psychomotor therapy, access to local gym  Physical health assessment and monitoring  Trauma-sensitive model of care |
| Cocchi, 2013^21^ | Education, awareness and anti-stigma campaigns for community organisations  Family psychoeducation, counselling or support  Mental health awareness and promotion campaigns for the general population, parents, and families  Training and mental health awareness for professionals working with young people |
| Cocchi, 2015a^22^ | Anti-stigma and youth-friendly community setting and service delivery  Family psychoeducation, counselling or support  Mental health awareness and promotion campaigns for the general population, parents, and families  Occupational or supportive therapy on vocational/occupational functioning  Educational support  Recreational therapy, activities or support  Recreational therapy, activities or support  Training and mental health awareness for professionals working with young people |
| Cocchi, 2015b^23^ | Education, awareness and anti-stigma campaigns for community organisations  Exercise/physical activity intervention, psychomotor therapy, access to local gym  Family psychoeducation, counselling or support  Individual or group psychoeducation  Occupational or supportive therapy on vocational/occupational functioning  Problem solving training  Service-user involvement on service development and delivery to include multicultural backgrounds  Training and mental health awareness for professionals working with young people |
| Coentre, 2020^24^ | Individual or group psychoeducation  Onsite vocational reintegration, including supported employment or IPS  Physical health assessment and monitoring |
| Formica, 2020^25^ | Individual or group psychoeducation  Problem solving training  Sleep hygiene or sleep interventions |
| Fusar-Poli, 2013^26^ | Comprehensive assessment of lifetime exposure to traumatic events  Education, awareness and anti-stigma campaigns for community organisations  Presence in catchment area with high levels of economic inequality, unemployment, and homelessness  Presence in catchment area with high levels of ethnic minorities  Presence in catchment area with high levels of social deprivation  Presence in a prison setting  Psychosocial support on social relationships and functioning  Recovery-oriented model of service with focus on social and role functional support  Sleep hygiene or sleep interventions |
| Fusar-Poli, 2014^27^ | Individual or group psychoeducation |
| Fusar-Poli, 2019^28^ | Education, awareness and anti-stigma campaigns for community organisations  Family psychoeducation, counselling or support  Individual or group psychoeducation  Mental health awareness and promotion campaigns for the general population, parents, and families  Occupational or supportive therapy on vocational/occupational functioning  Physical health assessment and monitoring  Psychosocial support on social relationships and functioning  Educational support  Recovery-oriented model of service with focus on social and role functional support  Service-user involvement on service development and delivery to include multicultural backgrounds  Service-users involvement for the promotion of mental health literacy  Training and mental health awareness for professionals working with young people |
| Fusar-Poli, 2020^29^ | Community engagement projects focused on asylum seekers and refugees  Community engagement projects focused on young people from ethnic minorities  Education, awareness and anti-stigma campaigns for community organisations  Family psychoeducation, counselling or support  Mental health awareness and promotion campaigns for the general population, parents, and families  Service-user involvement on service development and delivery to include multicultural backgrounds  Service-users involvement for the promotion of mental health literacy  Training and mental health awareness for professionals working with young people |
| Gaspar, 2018^30^ | Family psychoeducation, counselling or support  Mental health awareness and promotion campaigns for the general population, parents, and families  Physical health assessment and monitoring  Psychosocial support on social relationships and functioning  Training and mental health awareness for professionals working with young people |
| Ghio, 2012^31^ | Drama, music/arts therapy  Education, awareness and anti-stigma campaigns for community organisations  Exercise/physical activity intervention, psychomotor therapy, access to local gym  Individual or group psychoeducation  Mental health awareness and promotion campaigns for the general population, parents, and families  Occupational or supportive therapy on vocational/occupational functioning  Problem solving training  Service-user involvement on service development and delivery to include multicultural backgrounds  Training and mental health awareness for professionals working with young people |
| Green, 2011^32^ | Psychosocial support with housing and accommodation |
| Janssen, 2020^33^ | Exercise/physical activity intervention, psychomotor therapy, access to local gym  Family psychoeducation, counselling or support |
| Joa, 2015^34^ | Family psychoeducation, counselling or support  Mental health awareness and promotion campaigns for the general population, parents, and families  Recovery-oriented model of service with focus on social and role functional support  Training and mental health awareness for professionals working with young people |
| Joa, 2021^35^ | Education, awareness and anti-stigma campaigns for community organisations  Family psychoeducation, counselling or support  Mental health awareness and promotion campaigns for the general population, parents, and families  Recovery-oriented model of service with focus on social and role functional support  Training and mental health awareness for professionals working with young people |
| Katsura, 2014^36^ | Family psychoeducation, counselling or support  Mental health awareness and promotion campaigns for the general population, parents, and families  Training and mental health awareness for professionals working with young people |
| Kim, 2020^37^ | Anti-stigma and youth-friendly community setting and service delivery  Diet and healthy eating intervention or advice  Exercise/physical activity intervention, psychomotor therapy, access to local gym  Family psychoeducation, counselling or support  Flexible outreach approach to enhance engagement of young people  Individual or group psychoeducation |
| Kollias, 2016^38^ | Individual or group psychoeducation  Occupational or supportive therapy on vocational/occupational functioning  Psychosocial support on social relationships and functioning  Training and mental health awareness for professionals working with young people |
| Kollias, 2020^39^ | Coping strategies on group setting  Drama, music/arts therapy  Family psychoeducation, counselling or support  Occupational or supportive therapy on vocational/occupational functioning  Recovery-oriented model of service with focus on social and role functional support |
| Kotlicka-Antczak, 2015^40^ | Mental health awareness and promotion campaigns for the general population, parents, and families  Training and mental health awareness for professionals working with young people |
| Kotlicka-Antczak, 2016^41^ | Mental health awareness and promotion campaigns for the general population, parents, and families |
| Kotlicka-Antczak, 2020^42^ | Family psychoeducation, counselling or support  Occupational or supportive therapy on vocational/occupational functioning  Psychosocial support on social relationships and functioning  Educational support  Psychosocial support with housing and accommodation |
| Kwon, 2012^43^ | Family psychoeducation, counselling or support |
| Leuci, 2019^44^ | Family psychoeducation, counselling or support  Occupational or supportive therapy on vocational/occupational functioning  Onsite vocational reintegration, including supported employment or IPS  Psychosocial support on social relationships and functioning  Recovery-oriented model of service with focus on social and role functional support |
| Louza, 2008^45^ | Mental health awareness and promotion campaigns for the general population, parents, and families |
| Lynch 2016^46^ | Mental health awareness and promotion campaigns for the general population, parents, and families  Training and mental health awareness for professionals working with young people |
| McFarlane, 2012^47^ | Assessment of vocational history, goals and engagement, and cognitive functioning  Family psychoeducation, counselling or support  Onsite vocational reintegration, including supported employment or IPS  Presence in catchment area with high levels of ethnic minorities  Educational support |
| McFarlane, 2010^48^ | Education, awareness and anti-stigma campaigns for community organisations  Flexible outreach approach to enhance engagement of young people  Mental health awareness and promotion campaigns for the general population, parents, and families  Training and mental health awareness for professionals working with young people |
| Meneghelli, 2010^49^ | Education, awareness and anti-stigma campaigns for community organisations  Family psychoeducation, counselling or support  Mental health awareness and promotion campaigns for the general population, parents, and families  Occupational or supportive therapy on vocational/occupational functioning  Problem solving training  Educational support  Recreational therapy, activities or support  Training and mental health awareness for professionals working with young people |
| Meneghelli, 2020^50^ | Family psychoeducation, counselling or support  Individual or group psychoeducation  Occupational or supportive therapy on vocational/occupational functioning  Problem solving training  Psychosocial support on social relationships and functioning  Educational support  Recreational therapy, activities or support |
| Michel, 2021^51^ | Assessment of vocational history, goals and engagement, and cognitive functioning  Comprehensive assessment of lifetime exposure to traumatic events  Family psychoeducation, counselling or support  Individual or group psychoeducation |
| Belvederi Murri, 2020^16^ | Family psychoeducation, counselling or support  Onsite vocational reintegration, including supported employment or IPS  Psychosocial support on social relationships and functioning  Training and mental health awareness for professionals working with young people |
| Oppetit, 2016^52^ | Diet and healthy eating intervention or advice  Family psychoeducation, counselling or support  Individual or group psychoeducation  Mental health awareness and promotion campaigns for the general population, parents, and families  Psychosocial support on social relationships and functioning  Educational support  Training and mental health awareness for professionals working with young people |
| Ortega, 2020^53^ | Comprehensive assessment of lifetime exposure to traumatic events |
| Parabiaghi, 2019^54^ | Anti-stigma and youth-friendly community setting and service delivery  Education, awareness and anti-stigma campaigns for community organisations  Recreational therapy, activities or support |
| Pelizza, 2019^55^ | Family psychoeducation, counselling or support  Psychosocial support on social relationships and functioning  Recovery-oriented model of service with focus on social and role functional support |
| Pelizza, 2020^56^ | Family psychoeducation, counselling or support  Recovery-oriented model of service with focus on social and role functional support |
| Penno, 2017^57^ | Intensive networking with local/community stakeholders  Physical health assessment and monitoring |
| Phillips, 2002^58^ | Education, awareness and anti-stigma campaigns for community organisations  Intensive networking with local/community stakeholders  Mental health awareness and promotion campaigns for the general population, parents, and families  Psychosocial support with housing and accommodation  Training and mental health awareness for professionals working with young people |
| Poletti, 2020^59^ | Family psychoeducation, counselling or support  Recovery-oriented model of service with focus on social and role functional support |
| Power, 2007^60^ | Anti-stigma and youth-friendly community setting and service delivery  Family psychoeducation, counselling or support  Onsite vocational reintegration, including supported employment or IPS  Presence in catchment area with high levels of economic inequality, unemployment, and homelessness  Presence in catchment area with high levels of ethnic minorities  Presence in catchment area with high levels of social deprivation  Educational support  Training and mental health awareness for professionals working with young people |
| Pruessner, 2015^61^ | Diet and healthy eating intervention or advice  Education, awareness and anti-stigma campaigns for community organisations  Exercise/physical activity intervention, psychomotor therapy, access to local gym  Family psychoeducation, counselling or support  Occupational or supportive therapy on vocational/occupational functioning  Psychosocial support on social relationships and functioning  Recreational therapy, activities or support |
| Quijada, 2010^62^ | Education, awareness and anti-stigma campaigns for community organisations |
| Rao, 2013^63^ | Education, awareness and anti-stigma campaigns for community organisations  Family psychoeducation, counselling or support  Mental health awareness and promotion campaigns for the general population, parents, and families  Psychosocial support on social relationships and functioning  Educational support  Recovery-oriented model of service with focus on social and role functional support  Service-user involvement on service development and delivery to include multicultural backgrounds  Training and mental health awareness for professionals working with young people |
| Riecher-Rossler, 2007^64^ | Mental health awareness and promotion campaigns for the general population, parents, and families  Training and mental health awareness for professionals working with young people |
| Ruff, 2012^65^ | Education, awareness and anti-stigma campaigns for community organisations  Intensive networking with local/community stakeholders  Mental health awareness and promotion campaigns for the general population, parents, and families  Educational support  Training and mental health awareness for professionals working with young people |
| Schultze‑Lutter, 2009^66^ | Education, awareness and anti-stigma campaigns for community organisations  Mental health awareness and promotion campaigns for the general population, parents, and families  Training and mental health awareness for professionals working with young people |
| Selvendra, 2014^67^ | Family psychoeducation, counselling or support  Occupational or supportive therapy on vocational/occupational functioning  Physical health assessment and monitoring |
| Simon, 2012^68^ | Mental health awareness and promotion campaigns for the general population, parents, and families  Training and mental health awareness for professionals working with young people |
| Stain, 2017^69^ | Family psychoeducation, counselling or support  Individual or group psychoeducation |
| Tang, 2010^70^ | Mental health awareness and promotion campaigns for the general population, parents, and families |
| Tay, 2015^71^ | Individual or group psychoeducation  Intensive networking with local/community stakeholders  Psychosocial support on social relationships and functioning  Educational support  Recovery-oriented model of service with focus on social and role functional support |
| Theodoridou 2014^72^ | Mental health awareness and promotion campaigns for the general population, parents, and families  Training and mental health awareness for professionals working with young people |
| Tiffin, 2007^73^ | Diet and healthy eating intervention or advice  Individual or group psychoeducation  Intensive networking with local/community stakeholders  Life/practical skills training  Presence in catchment area with high levels of social deprivation  Educational support  Psychosocial support with housing and accommodation |
| Tognin, 2019^74^ | Assessment of vocational history, goals and engagement, and cognitive functioning  Intensive networking with local/community stakeholders  Occupational or supportive therapy on vocational/occupational functioning  Occupational or supportive therapy on vocational/occupational functioning  Onsite vocational reintegration, including supported employment or IPS |
| Ventura, 2021^75^ | Cognitive remediation training  Family psychoeducation, counselling or support  Individual or group psychoeducation  Training and mental health awareness for professionals working with young people |
| Wong, 2008^76^ | Mental health awareness and promotion campaigns for the general population, parents, and families |
| Wong, 2012^77^ | Mental health awareness and promotion campaigns for the general population, parents, and families |
| Yang, 2020^78^ | Anti-stigma and youth-friendly community setting and service delivery  Exercise/physical activity intervention, psychomotor therapy, access to local gym  Family psychoeducation, counselling or support  Inclusiveness of LGBT+ populations  Recovery-oriented model of service with focus on social and role functional support |
| Yung, 1998^79^ | Anti-stigma and youth-friendly community setting and service delivery  Education, awareness and anti-stigma campaigns for community organisations  Training and mental health awareness for professionals working with young people |

# **eFigure 1.** Services, networks, and surveys included in the systematic review


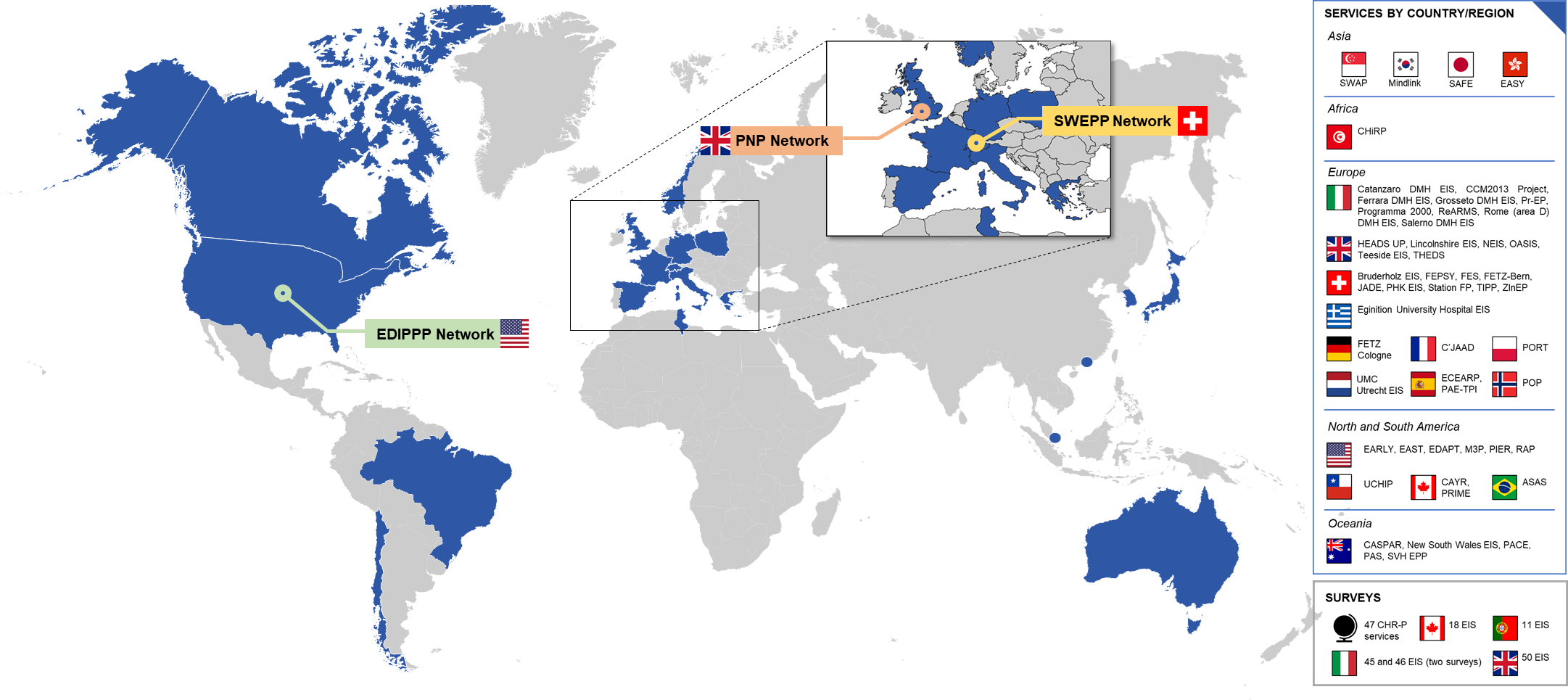
*Legend: ASAS, Evaluation and Follow-up of Adolescents and Young Adults in São Paulo; CASPAR, Comprehensive Assessment Service for Psychosis and At Risk; CAYR, Clinic for Assessment of Youth at Risk; CCM, National Centre for Disease Prevention and Control; CHiRP, clinical high-risk program of Razi Hospital; CHR-P, clinical high-risk of psychosis; C’JAAD, Evaluation Centre for Adolescents and Young Adults; DMH, Department of Mental Health; EARLY, Early Assessment and Resource Linkage for Youth; EAST, Early Assessment and Support Team;* *EASY, Early Assessment Service for Young people with psychosis; EDAPT, Early Detection and Preventive Treatment; ECEARP, Care Equipment for At-Risk of Psychosis Patients; EDIPPP, Early Detection Intervention and Prevention of Psychosis Program; EIS, early intervention service; FEPSY, Basel early-detection-of-psychosis study; FETZ, Cologne Early Recognition and Intervention Centre for mental crises; HEADS UP, City & Hackney At-Risk Mental State Service; M3P, Michigan Prevents Prodromal Progression; NEIS, Newham Early Intervention Service; OASIS, Outreach and Support in South London; PACE, Personal Assessment and Crisis Evaluation clinic; PAE-TPI, Early Psychotic Disorder Care Programmes; PAS, Psychological Assistance Service; PHK, Psychiatric Hospital Königsfelden; PIER, Portland Identification and Early Referral; PNP, Pan-London Network for Psychosis Prevention; POP, Prevention of Psychosis study; PORT, Programme of Recognition and Therapy; Pr-EP, Parma—Early Psychosis programme; PRIME, Toronto Prevention through Risk Identification, Management and Education; RAP, Recognition and Prevention programme; ReARMS, Reggio Emilia At-Risk Mental States programme; SAFE, Sendai ARMS and first episode clinic; Station FP, Psychiatric Hospital Münsterlinger Early Psychosis Outpatient Service; SVH EPP, St Vincent’s Hospital early psychosis programme; SWAP, Support for Wellness Achievement Programme; SWEPP, Swiss Early Psychosis Project; THEDS, Tower Hamlets Early Intervention Service; TIPP, Treatment and early Intervention in Psychosis Programme; UCHIP, University of Chile High-risk Intervention Program; UMC, University Medical Centre.*

# **eFigure 2.** Mental health literacy promotion materials at the OASIS service

The following 5 figures represent mental health literacy promotion material developed at the Outreach and Support in South London (OASIS) CHR-P service (<https://www.meandmymind.nhs.uk/>). Materials are authored by Jennifer Harries, member of the OASIS team.

*Fig. 2.a. Hearing voices*


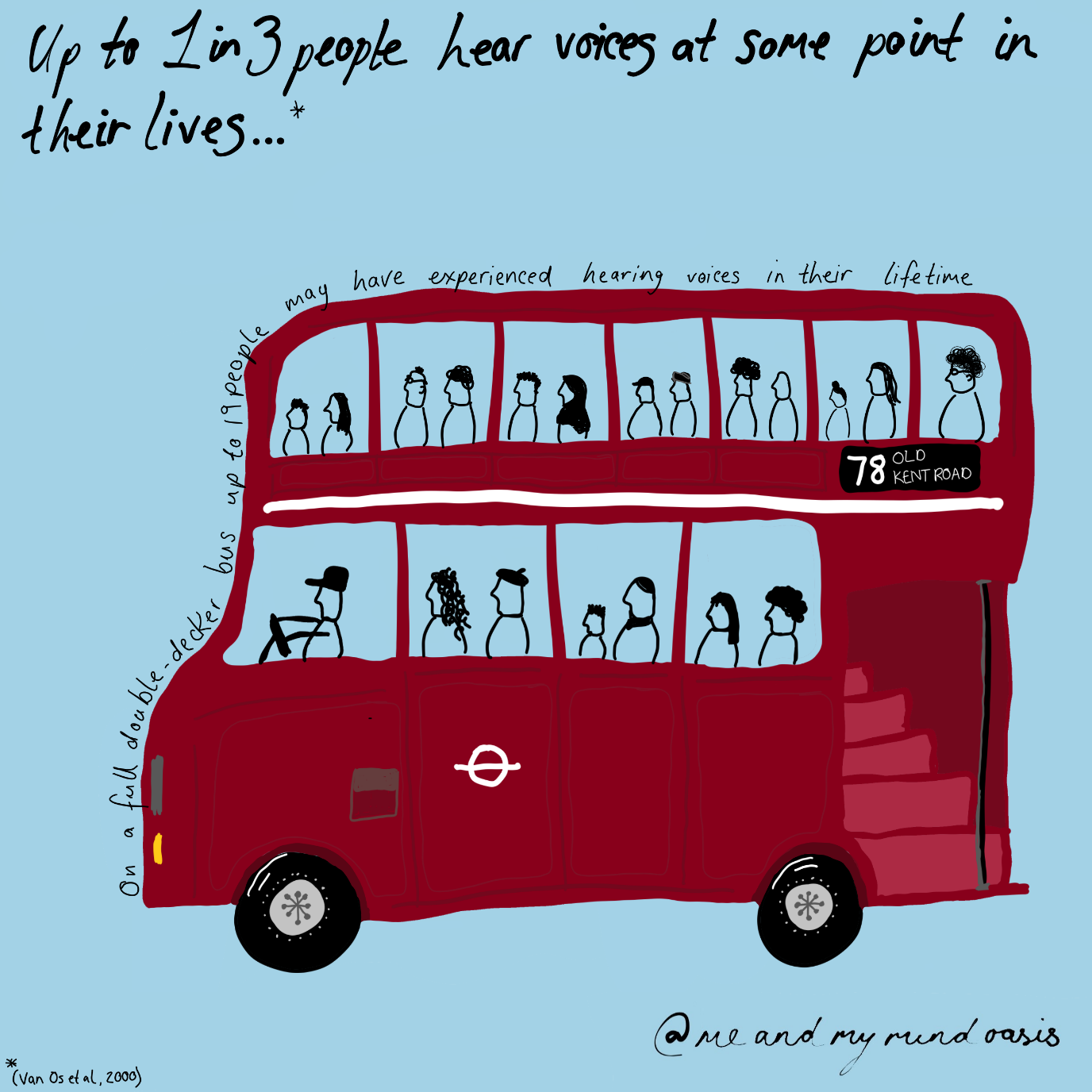


*Fig. 2.b. Coping with unusual experiences*


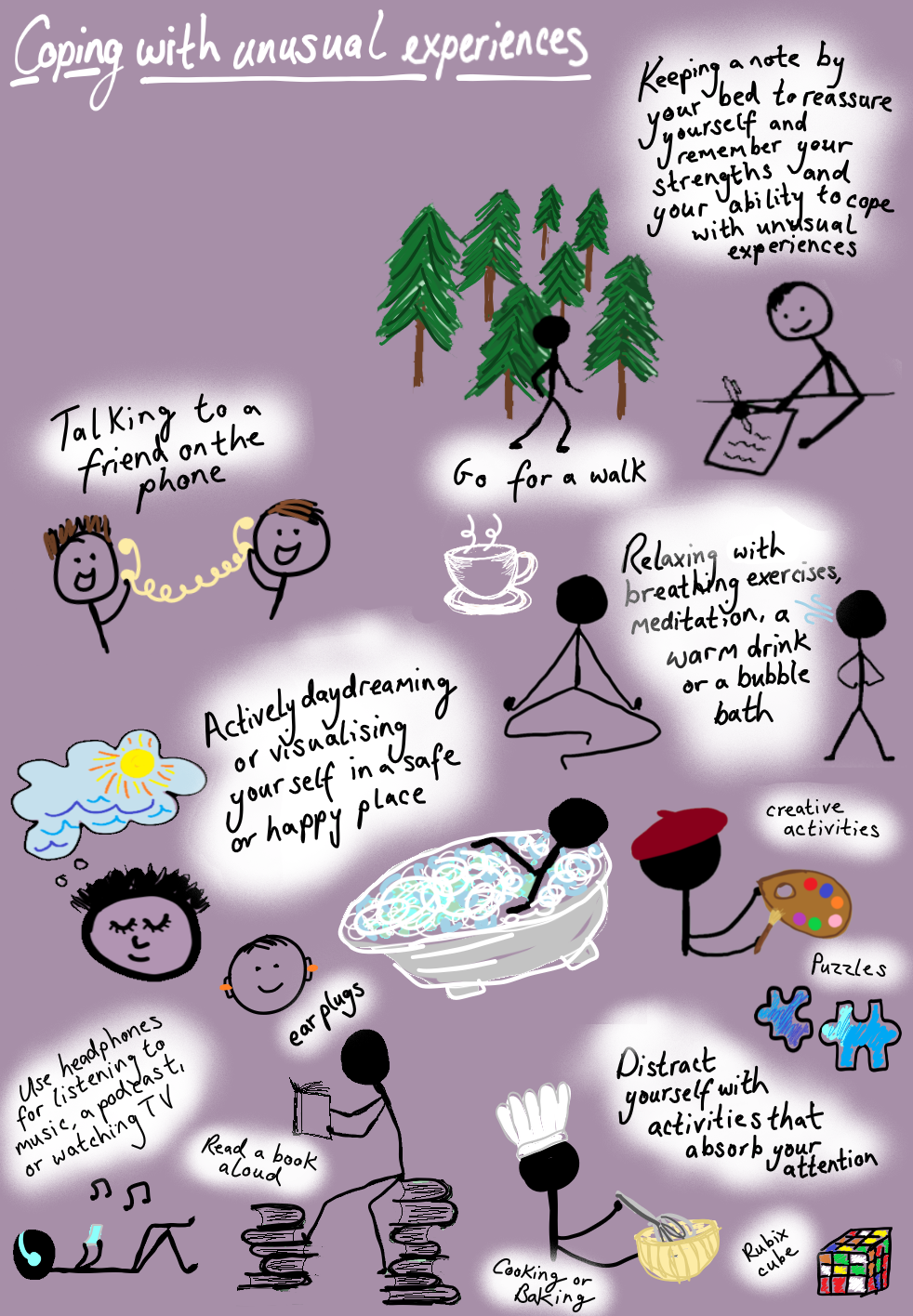


*Fig. 2.c. Looking after myself*


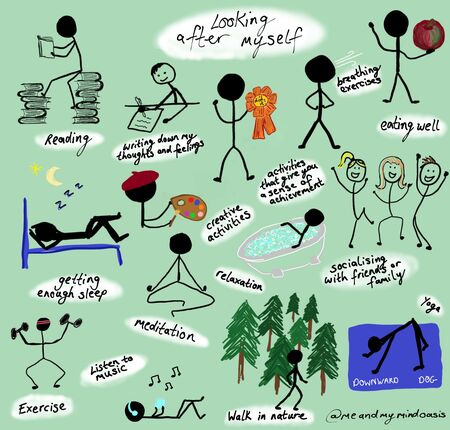


*Fig. 2.d. Unusual experiences*


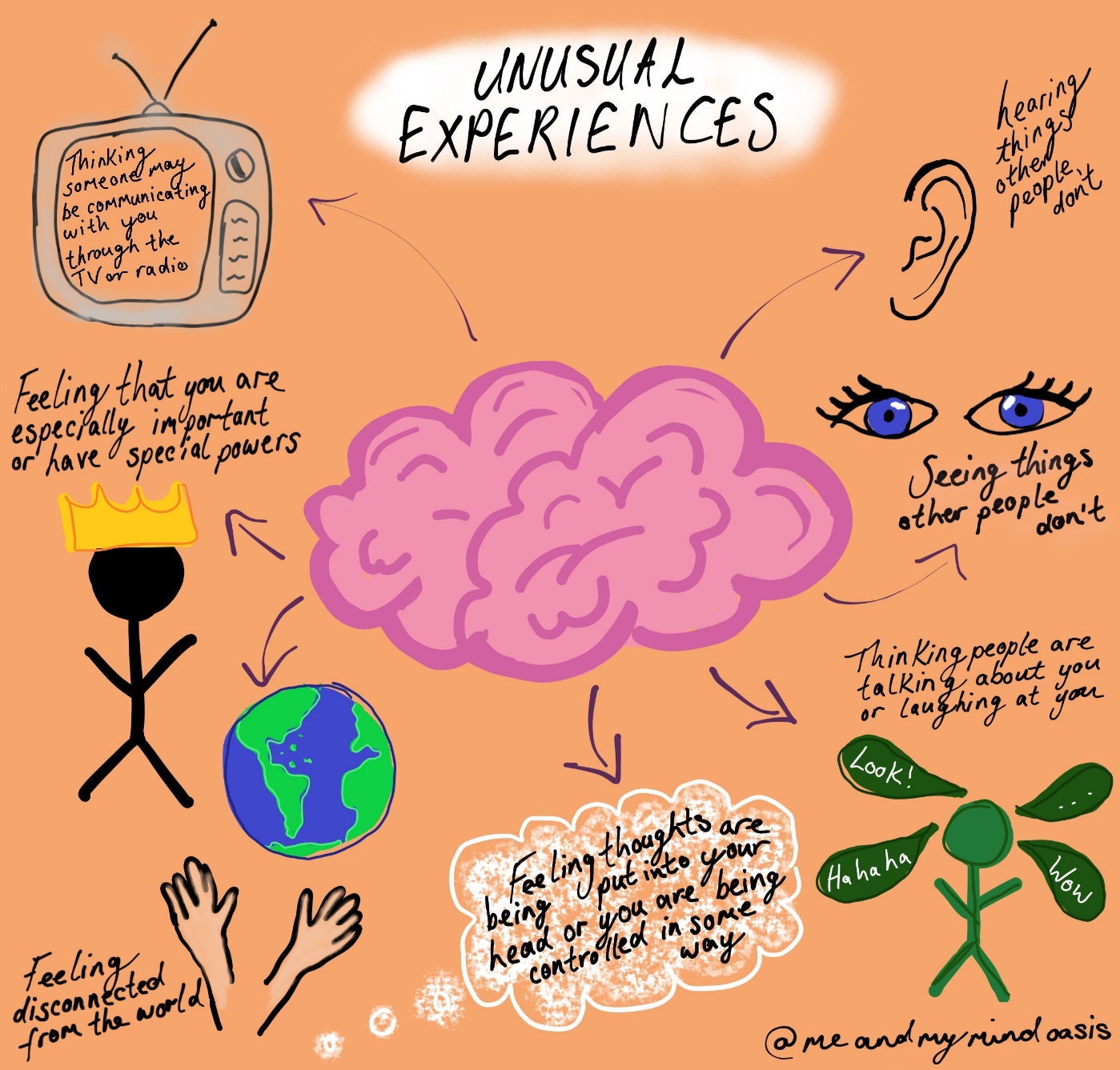


*Fig. 2.e. Ups and downs of mental health*


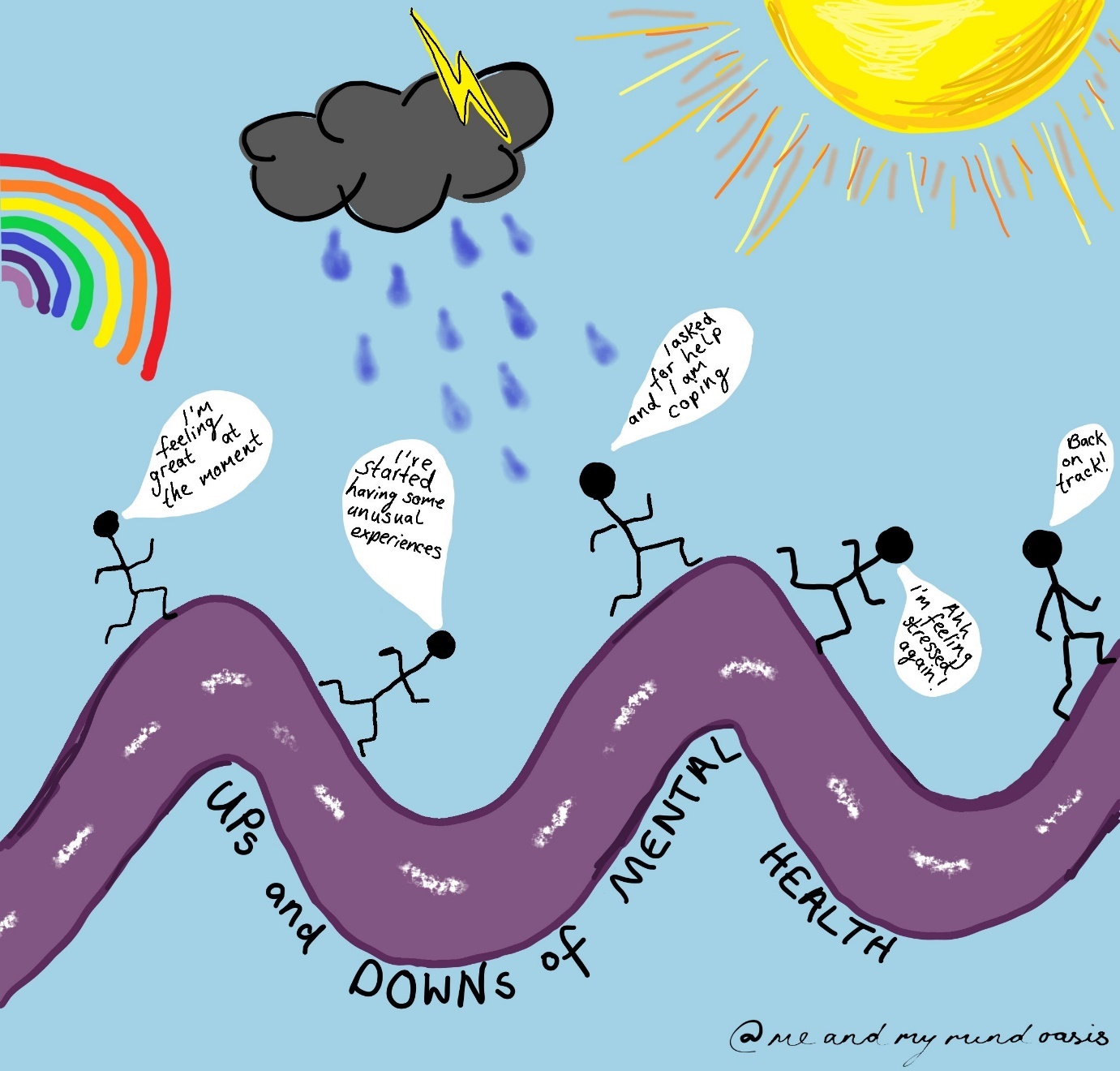


**REFERENCES**

1 Yung AR, Yung AR, Pan Yuen H, Mcgorry PD, Phillips LJ, Kelly D et al. Mapping the onset of psychosis: the Comprehensive Assessment of At-Risk Mental States. Aust New Zeal J Psychiatry 2005; 39: 964–971.

2 Fusar-Poli P, Cappucciati M, Rutigliano G, Lee TY, Beverly Q, Bonoldi I et al. Towards a standard psychometric diagnostic interview for subjects at ultra high risk of psychosis: CAARMS versus SIPS. Psychiatry J 2016; 2016: 1–11.

3 McGlashan TH, Walsh BC, Woods SW. The psychosis-risk syndrome: handbook for diagnosis and follow-up. Oxford University Press: Oxford, 2010.

4 Vollmer-Larsen A, Handest P, Parnas J. Reliability of measuring anomalous experience: The Bonn Scale for the Assessment of Basic Symptoms. Psychopathology 2007; 40: 345–348.

5 Riecher-Rössler A, Aston J, Ventura J, Merlo M, Borgwardt S, Gschwandtner U et al. [The Basel Screening Instrument for Psychosis (BSIP): development, structure, reliability and validity]. Fortschr Neurol Psychiatr 2008; 74: 207–216.

6 Fux L, Walger P, Schimmelmann BG, Schultze-Lutter F. The Schizophrenia Proneness Instrument, Child and Youth version (SPI-CY): practicability and discriminative validity. Schizophr Res 2013; 146: 69–78.

7 Kay SR, Fiszbein A, Opler LA. The positive and negative syndrome scale (PANSS) for schizophrenia. Schizophr Bull 1987; 13: 261–276.

8 N C. Andreasen. The scale for the assessment of negative symptoms (SANS). Conceptual and Theoretical Foundations. Br J Psychiatry 1984; 155: 49–52.

9 Overall J, Gorham D. The Brief Psychiatric Rating Scale (BPRS): recent developments in ascertainment and scaling. Psychopharmacol Bull 1988; 24: 99–97.

10 Haefner H, Bechdolf A, Klosterkotter J, Maurer K. Early detection and intervention in psychosis. A practice handbook. Stuttgart, 2011.

11 Yung AR, Stanford C, Cosgrave E, Killackey E, Phillips L, Nelson B et al. Testing the ultra high risk (prodromal) criteria for the prediction of psychosis in a clinical sample of young people. Schizophr Res 2006; 84: 57–66.

12 Yung AR, Phillips LJ, Pan H, Francey SM, Mcfarlane CA, Hallgren M et al. Psychosis prediction: 12-month follow up of a high-risk (‘prodromal’) group. Schizophr Res 2003; 60: 21–32.

13 Fusar-Poli P, Salazar de Pablo G, De Micheli A, Nieman DH, Correll CU, Kessing LV et al. What is good mental health? A scoping review. Eur Neuropsychopharmacol 2020; 31: 33–46.

14 Adamson V, Barrass E, McConville S, Irikok C, Taylor K, Pitt S et al. Implementing the access and waiting time standard for early intervention in psychosis in the United Kingdom: an evaluation of referrals and post-assessment outcomes over the first year of operation. Early Interv Psychiatry 2018; 12: 979–986.

15 Addington J, Epstein I, Reynolds A, Furimsky I, Rudy L, Mancini B et al. Early detection of psychosis: finding those at clinical high risk. Early Interv Psychiatry 2008; 2: 147–153.

16 Belvederi Murri M, Bertelli R, Carozza P, Berardi L, Cantarelli L, Croce E et al. First-episode psychosis in the Ferrara Mental Health Department: incidence and clinical course within the first 2 years. Early Interv Psychiatry 2020; 1–11.

17 Bertulies-Esposito B, Nolin M, Iyer SN, Malla A, Tibbo P, Otter N et al. Où en sommes-nous? An overview of successes and challenges after 30 years of early intervention services for psychosis in Quebec. Can J Psychiatry 2020. doi:10.1177/0706743719895193.

18 Broome MR, Woolley JB, Johns LC, Valmaggia LR, Tabraham P, Gafoor R et al. Outreach and support in south London (OASIS): implementation of a clinical service for prodromal psychosis and the at risk mental state. Eur Psychiatry 2005; 20: 372–378.

19 Carr V, Halpin S, Lau N, O’brien S, Beckmann J, Lewin T. A risk factor screening and assessment protocol for schizophrenia and related psychosis. Aust N Z J Psychiatry 2000; 34: S170–S180.

20 Coates D, Wright L, Moore T, Pinnell S, Merillo C, Howe D. The psychiatric, psychosocial and physical health profile of young people with early psychosis: data from an early psychosis intervention service. Child Youth Serv 2019; 40: 93–115.

21 Cocchi A, Meneghelli A, Erlicher A, Pisano A, Cascio MT, Preti A. Patterns of referral in first-episode schizophrenia and ultra high-risk individuals: results from an early intervention program in Italy. Soc Psychiatry Psychiatr Epidemiol 2013; 48: 1905–1916.

22 Cocchi A, Balbi A, Corlito G, Ditta G, Di Munzio W, Nicotera M et al. Early intervention in psychosis: a feasibility study financed by the Italian Center on Control of Maladies. Early Interv Psychiatry 2015; 9: 163–171.

23 Cocchi A, Cavicchini A, Collavo M, Ghio L, Macchi S, Meneghelli A et al. Implementation and development of early intervention in psychosis services in Italy: a national survey promoted by the Associazione Italiana Interventi Precoci nelle Psicosi. Early Interv Psychiatry 2015; 12: 37–44.

24 Coentre R, Levy P. Early intervention in psychosis: The first national survey in Portugal. Schizophr Res 2020; 218: 298–299.

25 Formica MJC, Phillips LJ, Hartmann JA, Yung AR, Wood SJ, Lin A et al. Has improved treatment contributed to the declining rate of transition to psychosis in ultra-high-risk cohorts? Schizophr Res 2020. doi:10.1016/j.schres.2020.04.028.

26 Fusar-Poli P, Byrne M, Badger S, Valmaggia LR, McGuire PK. Outreach and support in South London (OASIS), 2001-2011: ten years of early diagnosis and treatment for young individuals at high clinical risk for psychosis. Eur Psychiatry 2013; 28: 315–326.

27 Fusar-Poli P, Frascarelli M, Valmaggia L, Byrne M, Stahl D, Rocchetti M et al. Antidepressant, antipsychotic and psychological interventions in subjects at high clinical risk for psychosis: OASIS 6-year naturalistic study. Psychol Med 2014; 45: 1327–1339.

28 Fusar-Poli P, Estradé A, Spencer TJ, Gupta S, Murguia-Asensio S, Eranti S et al. Pan-London Network for Psychosis-Prevention (PNP). Front Psychiatry 2019; 10: 1–10.

29 Fusar-Poli P, Spencer T, De Micheli A, Curzi V, Nandha S, McGuire P. Outreach and support in South-London (OASIS) 2001—2020: twenty years of early detection, prognosis and preventive care for young people at risk of psychosis. Eur Neuropsychopharmacol 2020; 39: 111–122.

30 Gaspar PA, Castillo RI, Maturana A, Villar MJ, Ulloa K, González G et al. Early psychosis detection program in Chile: a first step for the South American challenge in psychosis research. Early Interv Psychiatry 2018; 13: 328–334.

31 Ghio L, Natta W, Peruzzo L, Gotelli S, Tibaldi G, Ferrannini L. Process of implementation and development of early psychosis clinical services in Italy: a survey. Early Interv Psychiatry 2012; 6: 341–346.

32 Green CEL, McGuire PK, Ashworth M, Valmaggia LR. Outreach and Support in South London (OASIS). Outcomes of non-attenders to a service for people at high risk of psychosis: the case for a more assertive approach to assessment. Psychol Med 2011; 41: 243–250.

33 Janssen H, Maat A, Slot MIE, Scheepers F. Efficacy of psychological interventions in young individuals at ultra-high risk for psychosis: A naturalistic study. Early Interv Psychiatry 2020; 1–9.

34 Joa I, Gisselgård J, Brønnick K, McGlashan T, Johannessen JO. Primary prevention of psychosis through interventions in the symptomatic prodromal phase, a pragmatic Norwegian ultra high risk study. BMC Psychiatry 2015; 15: 1–9.

35 Joa I, Bjornestad J, Johannessen JO, Langeveld J, Stain HJ, Weibell M et al. Early detection of ultra high risk for psychosis in a Norwegian catchment area: the two year follow-up of the prevention of psychosis study. Front Psychiatry 2021; 12: 1–10.

36 Katsura M, Ohmuro N, Obara C, Kikuchi T, Ito F, Miyakoshi T et al. A naturalistic longitudinal study of at-risk mental state with a 2.4 year follow-up at a specialized clinic setting in Japan. Schizophr Res 2014; 158: 32–38.

37 Kim SW, Kim JK, Jhon M, Lee HJ, Kim H, Kim JW et al. Mindlink: A stigma-free youth-friendly community-based early-intervention centre in Korea. Early Interv Psychiatry 2020; 1–6.

38 Kollias C, Xenaki LA, Dimitrakopoulos S, Kosteletos I, Kontaxakis V, Stefanis N et al. Early psychosis intervention outpatient service of the 1st Psychiatric University Clinic in Athens: 3 Years of experience. Early Interv Psychiatry 2016; 12: 491–496.

39 Kollias K, Xenaki L, Vlachos I, Dimitrakopoulos S, Kosteletos I, Nianiakas N et al. The development of the Early Intervention in Psychosis (EIP) outpatient unit of Eginition University Hospital into an EIP Network K. 2020; 31: 177–182.

40 Kotlicka-Antczak M, Pawełczyk T, Rabe-Jabłońska J, Pawełczyk A. PORT (Programme of Recognition and Therapy): the first Polish recognition and treatment programme for patients with an at-risk mental state. Early Interv Psychiatry 2015; 9: 339–342.

41 Kotlicka-Antczak M, Pawełczyk T, Podgórski M, Żurner N, Karbownik MS, Pawełczyk A. Polish individuals with an at-risk mental state: demographic and clinical characteristics. Early Interv Psychiatry 2016; 12: 391–399.

42 Kotlicka-Antczak M, Podgórski M, Oliver D, Maric NP, Valmaggia L, Fusar-Poli P. Worldwide implementation of clinical services for the prevention of psychosis: the IEPA early intervention in mental health survey. Early Interv Psychiatry 2020; 14: 741–750.

43 Kwon JS, Byun MS, Lee TY, An SK. Early intervention in psychosis: insights from Korea. Asian J Psychiatr 2012; 5: 98–105.

44 Leuci E, Quattrone E, Pellegrini P, Pelizza L. The “Parma—Early Psychosis” program: general description and process analysis after 5 years of clinical activity. Early Interv Psychiatry 2019; : 1–9.

45 Louza MR, Azevedo Y, Macedo G, Gattaz W. An early psychosis research program in Sao Paulo, Brazil. Organization and implementation. Clin Neuropsychiatry J Treat Eval 2008; 5: 273–278.

46 Lynch S, McFarlane WR, Joly B, Adelsheim S, Auther A, Cornblatt BA et al. Early detection, intervention and prevention of psychosis program: community outreach and early identification at six U.S. sites. Psychiatr Serv 2016; 67: 510–516.

47 McFarlane WR, Cook WL, Downing D, Ruff A, Lynch S, Adelsheim S et al. Early detection, intervention, and prevention of psychosis program: rationale, design, and sample description. Adolesc Psychiatrye 2012; 2: 112–124.

48 McFarlane WR, Cook WL, Downing D, Verdi MB, Woodberry KA, Ruff A. Portland identification and early referral: a community-based system for identifying and treating youths at high risk of psychosis. Psychiatr Serv 2010; 61: 512–515.

49 Meneghelli A, Cocchi A, Preti A. ‘Programma2000’: a multi-modal pilot programme on early intervention in psychosis underway in Italy since 1999. Early Interv Psychiatry 2010; 4: 97–103.

50 Meneghelli A, Barbera S, Meliante M, Monzani E, Preti A, Cocchi A et al. Outcome at 2-year of treatment in first-episode psychosis patients who were enrolled in a specialized early intervention program. Psychiatry Res 2020; 291: 113200.

51 Michel C, Kaess M, Flückiger R, Büetiger JR, Schultze-Lutter F, Schimmelmann BG et al. The Bern Early Recognition and Intervention Centre for mental crisis (FETZ Bern)—An 8-year evaluation. Early Interv Psychiatry 2021; 1–13.

52 Oppetit A, Bourgin J, Martinez G, Kazes M, Mam-Lam-Fook C, Gaillard R et al. The C’JAAD: a French team for early intervention in psychosis in Paris. Early Interv Psychiatry 2016; 12: 243–249.

53 Ortega L, Montalvo I, Solé M, Creus M, Cabezas Á, Gutiérrez-Zotes A et al. Relationship between childhood trauma and social adaptation in a sample of young people attending an early intervention service for psychosis. Rev Psiquiatr Salud Ment 2020; 13: 131–139.

54 Parabiaghi A, Confalonieri L, Magnani N, Lora A, Butteri E, Prato K et al. Integrated programs for early recognition of severe mental disorders: recommendations from an Italian multicenter project. Front Psychiatry 2019; 10: 1–6.

55 Pelizza L, Azzali S, Paterlini F, Garlassi S, Scazza I, Chiri LR et al. The “Reggio Emilia At-Risk Mental States” program: a diffused, “liquid” model of early intervention in psychosis implemented in an Italian Department of Mental Health. Early Interv Psychiatry 2019; 13: 1513–1524.

56 Pelizza L, Azzali S, Garlassi S, Scazza I, Paterlini F, Chiri LR et al. Assessing aberrant salience in young community help-seekers with early psychosis: the approved Italian version of the Aberrant Salience Inventory. J Clin Psychol 2020. doi:10.1002/jclp.23059.

57 Penno SJ, Hamilton B, Petrakis M. Early intervention in psychosis: Health of the Nation Outcome Scales (HoNOS) outcomes from a five-year prospective study. Arch Psychiatr Nurs 2017; 31: 553–560.

58 Phillips L, Leicester S, O’Dwyer L, Francey S, Koutsogiannis J, Abdel-Baki A et al. The PACE Clinic: identification and management of young people at “ultra” high risk of psychosis. J Psychiatr Pract 2002; 8: 255–269.

59 Poletti M, Pelizza L, Azzali S, Paterlini F, Garlassi S, Scazza I et al. Overcoming the gap between child and adult mental health services: the Reggio Emilia experience in an early intervention in psychosis program. Early Interv Psychiatry 2020; 1–10.

60 Power P, McGuire P, Iacoponi E, Garety P, Morris E, Valmaggia L et al. Lambeth Early Onset (LEO) and Outreach & Support in South London (OASIS) service. Early Interv Psychiatry 2007; 1: 97–103.

61 Pruessner M, Faridi K, Shah J, Rabinovitch M, Iyer S, Abadi S et al. The Clinic for Assessment of Youth at Risk (CAYR): 10 years of service delivery and research targeting the prevention of psychosis in Montreal, Canada. Early Interv Psychiatry 2015; 11: 177–184.

62 Quijada Y, Tizón JL, Artigue J, Parra B. At-risk mental state (ARMS) detection in a community service center for early attention to psychosis in Barcelona. Early Interv Psychiatry 2010; 4: 257–262.

63 Rao S, Pariyasami S, Tay S., Lim L., Yuen S, Poon L. et al. Support for Wellness Achievement Programme (SWAP): a service for individuals with at-risk mental state in Singapore. Ann Acad Med Singap 2013; : 552–555.

64 Riecher-Rössler A, Gschwandtner U, Aston J, Borgwardt S, Drewe M, Fuhr P et al. The Basel early-detection-of-psychosis (FEPSY)-study - Design and preliminary results. Acta Psychiatr Scand 2007; 115: 114–125.

65 Ruff A, McFarlane WR, Downing D, Cook W, Woodberry K. A community outreach and education model for early identification of mental illness in young people. Adolesc Psychiatrye 2012; 2: 140–145.

66 Schultze-Lutter F, Ruhrmann S, Klosterkötter J. Early detection of psychosis - Establishing a service for persons at risk. Eur Psychiatry 2009; 24: 1–10.

67 Selvendra A, Baetens D, Trauer T, Petrakis M, Castle D. First episode psychosis in an adult area mental health service - A closer look at early and late-onset first episode psychosis. Australas Psychiatry 2014; 22: 235–241.

68 Simon AE, Theodoridou A, Schimmelmann B, Schneider R, Conus P. The Swiss Early Psychosis Project SWEPP: a national network. Early Interv Psychiatry 2012; 6: 106–111.

69 Stain HJ, Mawn L, Common S, Pilton M, Thompson A. Research and practice for ultra-high risk for psychosis: a national survey of early intervention in psychosis services in England. Early Interv Psychiatry 2017; 1–6.

70 Tang JYM, Wong GHY, Hui CLM, Lam MML, Chiu CPY, Chan SKW et al. Early intervention for psychosis in Hong Kong - the EASY programme. Early Interv Psychiatry 2010; 4: 214–219.

71 Tay SA, Yuen S, Lim LK, Pariyasami S, Rao S, Poon LY et al. Support for Wellness Achievement Programme (SWAP): clinical and demographic characteristics of young people with at-risk mental state in Singapore. Early Interv Psychiatry 2015; 9: 516–522.

72 Theodoridou A, Heekeren K, Dvorsky D, Metzler S, Franscini M, Haker H et al. Early recognition of high risk of bipolar disorder and psychosis: an overview of the ZInEP ‘early recognition’ study. Front Public Heal 2014; 2: 1–8.

73 Tiffin PA, Hudson S. Early intervention in the real world: an early intervention in psychosis Service for adolescents. Early Interv Psychiatry 2007; 1: 212–218.

74 Tognin S, Grady L, Ventura S, Valmaggia L, Sear V, McGuire P et al. The provision of Education and employment support at the outreach and support in South London (OASIS) service for people at clinical high risk for psychosis. Front Psychiatry 2019; 10: 1–9.

75 Ventura J, Jouini L, Aissa A, Larnaout A, Nefzi R, Ghazzai M et al. Establishing a clinical high-risk program in Tunisia, North Africa: a pilot study in early detection and identification. Early Interv Psychiatry 2021; 1–7.

76 Wong GH, Hui CL, Chiu CP, Lam M, Chung DW, Tso S et al. Early detection and intervention for psychosis in Hong Kong: experience of a population-based intervention programme. Clin Neuropsychiatry J Treat Eval 2008; 5: 286–289.

77 Wong GHY, Hui CLM, Tang JYM, Chang WC, Chan SKW, Xu JQ et al. Early intervention for psychotic disorders: real-life implementation in Hong Kong. Asian J Psychiatr 2012; 5: 68–72.

78 Yang R, Curtis J, Jensen C, Levy P, Chown K, Lappin JM. Detection and intervention in emerging youth mental health issues: outcomes from the first year of the CASPAR service. Early Interv Psychiatry 2020; 1–7.

79 Yung AR, Phillips LJ, McGorry PD, Hallgren MA, McFarlane CA, Jackson HJ et al. Can we predict the onst of first-episode psychosis in a high-risk group? Int Clin Psychopharmacol 1998; 13: S23–S30.
